# A state-of-the-art review of the recent advances in exosome isolation and detection methods in viral infection

**DOI:** 10.1186/s12985-024-02301-5

**Published:** 2024-01-30

**Authors:** Amirsasan Gorgzadeh, Ahmad Nazari, Adnan Ali Ehsan Ismaeel, Diba Safarzadeh, Jawad A. K. Hassan, Saman Mohammadzadehsaliani, Hadis Kheradjoo, Pooneh Yasamineh, Saman Yasamineh

**Affiliations:** 1 Faculty of Pharmacy, Jondi Shapour University, Ahvaz, Iran; 2 Tehran University of Medical Sciences, Tehran, Iran; 3 Department of Pharmacy, Al-Noor University College, Mosul, Nineveh Iraq; 4 Vocational School of Health Service, Near East University, Nicosia, Cyprus; 5 National University of Science and Technology, Nasiriyah, Dhi Qar Iraq; 6 Ophthalmology Department, Buraimi Hospital, Buraimi, Oman; 7 Laboratory Department, Buraimi Hospital, Buraimi, Oman; 8 Young Researchers and Elite Club, Tabriz Branch, Islamic Azad University, Tabriz, Iran

**Keywords:** Exosome, Viral infection, Detection, Biomarkers, Purifications

## Abstract

Proteins, RNA, DNA, lipids, and carbohydrates are only some of the molecular components found in exosomes released by tumor cells. They play an essential role in healthy and diseased cells as messengers of short- and long-distance intercellular communication. However, since exosomes are released by every kind of cell and may be found in blood and other bodily fluids, they may one day serve as biomarkers for a wide range of disorders. In many pathological conditions, including cancer, inflammation, and infection, they play a role. It has been shown that the biogenesis of exosomes is analogous to that of viruses and that the exosomal cargo plays an essential role in the propagation, dissemination, and infection of several viruses. Bidirectional modulation of the immune response is achieved by the ability of exosomes associated with viruses to facilitate immunological escape and stimulate the body's antiviral immune response. Recently, exosomes have received a lot of interest due to their potential therapeutic use as biomarkers for viral infections such as human immunodeficiency virus (HIV), Hepatitis B virus (HBV), Hepatitis C virus (HCV), Epstein–Barr virus (EBV), and SARS-CoV-2. This article discusses the purification procedures and detection techniques for exosomes and examines the research on exosomes as a biomarker of viral infection.

**Graphical abstract:**

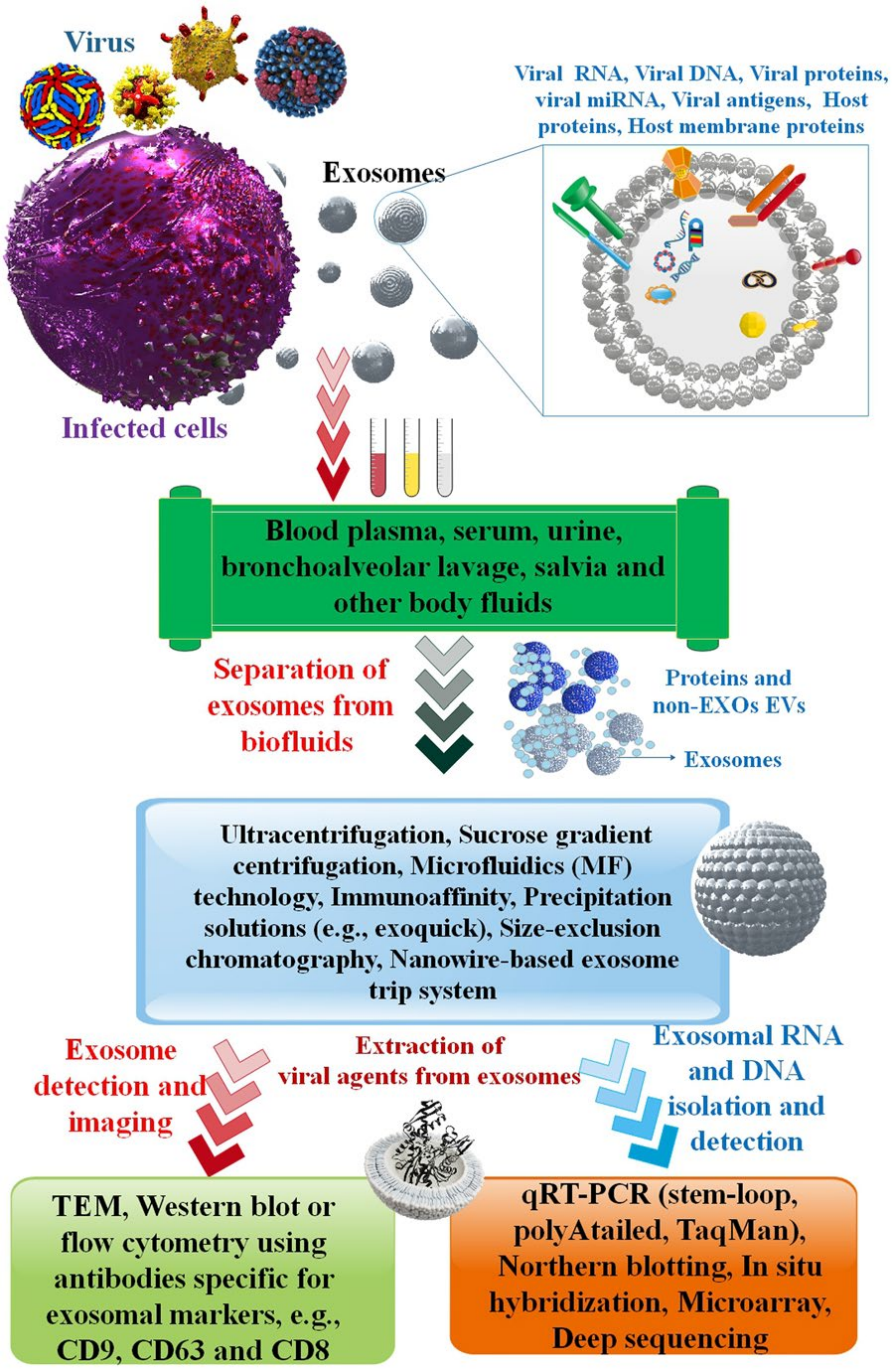

## Introduction

Almost all cell types produce lipid-bound vesicles called extracellular vehicles (EVs) into the extracellular lumen. EVs originate in the plasma membrane or endosomes. Based on their size, composition, biogenesis, and function, EVs are divided into three primary subtypes: exosomes (30–100 nm), microvesicles (100–1000 nm), and apoptotic bodies (500–4000 nm) [1]. Cerebrospinal fluid, saliva, blood, breast milk, ascetic fluid, amniotic fluid, seminal fluid, and urine are only some of the biological fluids shown to contain these EVs subtypes. At first, EVs were just thought of as junk that cells shed, but now we know better. EVs play a role in various physiological and pathological processes, including the immune response, neuron-glia communication, myelination, infection, and even cancer. One EV that has garnered much interest in the last several decades is the exosome, which has applications ranging from gene and medication delivery to prognostic and diagnostic biomarkers [2]. Exosomes are phospholipid nanocarriers derived from extracellular cells that act as signalosomes to deliver bioactive molecules to specific recipient cells for intercellular communication over short and long distances. Cargoes contained within exosomes, such as DNA, RNA, protein, lipid, carbohydrate, and other small molecules, can function in specific tissues to control cell proliferation, differentiation, migration, survival, gene expression, and cellular metabolism. Exosomes are involved in almost every illness studied [3–5]. Scientists are becoming more interested in exosomes, as well as their properties, functions, and analytical approaches. Recent advances in understanding the structural properties and functional uses of exosomes are reviewed. In addition, the current developments in exosome purification and testing procedures are highlighted. At last, the problems with exosome detection are laid forth. Prospects and challenges for future research into purification and quantitative detection approaches based on newly found properties of exosomes are discussed [6]. Numerous techniques have been developed through the investigation of the biochemical and physicochemical characteristics of exosomes; these include size exclusion chromatography (SEC), immunoaffinity capture, differential centrifugation, filtration centrifugation, density gradient centrifugation, and microfluidic technology. Currently, a standardized exosome isolation method is still lacking. Numerous techniques are used for storage, quality assurance, and purification. Exosome preparation yields variable results due to the dynamic homeostasis of the biological body; as a result, many methods have been developed to boost the exosome generation ratio, such as altering physical and chemical factors, increasing intracellular calcium concentration, drug stimulation, and gene overexpression. Additionally, exosomes are identified by their physicochemical characteristics, such as size and protein expression. Transmission electron microscopy (TEM), western blotting, nanoparticle tracking analysis (NTA), and protein concentration determination are commonly used to characterize exosomes. Years later, several issues still need to be urgently handled, including standard procedures for preparation and quality control and efficient quantification techniques for their simultaneous, complete, and transparent inclusion [7–10].

Exosomes are cell-derived, monolayer vesicles that serve as mediators of intercellular communication. It affects the body’s pathophysiology in conditions as diverse as cancer, inflammation, infection, and diagnosis. It has been shown that the biogenesis of exosomes is analogous to that of viruses and that the exosomal cargo plays a vital role in the propagation, dissemination, and infection of many viruses. Researchers investigate the potential of exosomes and their function in human immunodeficiency virus (HIV), Hepatitis B virus (HBV), Hepatitis C virus (HCV), and SARS-CoV-2 infection. These discoveries may lead to new treatments for viral infections and a decrease in illness incidence [11]. Infected cells secrete Exosomes, which may include viral DNA and RNA in addition to mRNA, microRNA, other forms of RNA, proteins, and even virions. Exosomes may deliver viral components to cells in organs and tissues that have not been infected. Through molecular and cellular processes involving Rab and endosomal sorting complex required for transport (ESCRT) proteins, viruses can infect cells and propagate through exosome release and subsequent viral infection. Exosomes have been demonstrated to have opposing impacts on the pathogenesis of viral infections, either attenuating or exacerbating illness progression. Exosomes may indicate infection severity in noninvasive diagnostics, while exosomes encapsulating biomolecules and medicines may be employed in treatment [12]. Prognosis and prevention of viral infections may benefit from understanding the central function of exosomes in disease manifestation and utilizing the same in therapies and diagnostics. This review examines exosomes and their function in the spread of illness, and their use in the treatment of viral infections. Additionally, diagnostic and prognostic data may be gleaned from detecting viral RNA, DNA, or virions. Patients' illness states may be evaluated using this data regularly. Developing therapeutic uses for exosomes requires a knowledge of how exosomes boost viral infectivity. Exosomes have been related to several viruses, including Epstein–Barr virus (EBV), hepatitis, Ebola, and COVID-19 [13, 14]. In this review, the characteristics of exosomes, their extraction and purification, and their detection methods were all explored in this overview. Additionally, we review the literature on exosomes as a biomarker in viral infection.

## Characterization of exosomes and biogenesis

The three steps involved in exosome biogenesis are endocytic vesicle creation within the cell, multivesicular bodies (MVB) production, and exosome release. Fusion of the MVB with the lysosome leads to its degradation as well. Exosomes, like host cells, have membranes made of a lipid bilayer and include all of the host cell's known molecular components, such as proteins, DNAs, RNAs, lipids, and metabolites [15]. Eukaryotic and prokaryotic cells produce EVs physiologically and in response to acquired disorders. Exosomes include a wide variety of components because of the plasma membrane's sequential invagination, which leads to the creation of MVBs that may intersect with other intracellular vesicles and organelles. Disease progression or suppression may result from exosome-mediated responses. Targeted distribution of therapeutic payloads, such as small interfering RNAs, antisense oligonucleotides, chemotherapeutic drugs, and immunological modulators, is possible via the engineering of exosomes. Pharmacokinetic features of exosomes may be affected by their lipid and protein content; their natural elements may contribute to increased bioavailability and reduced adverse effects [16]. Exosomes can potentially help in illness diagnostics in addition to their therapeutic use. They have been found in every known body fluid, and liquid biopsies may be used to quickly determine the complicated payload carried by exosomes [17].

**Fig. 1:**
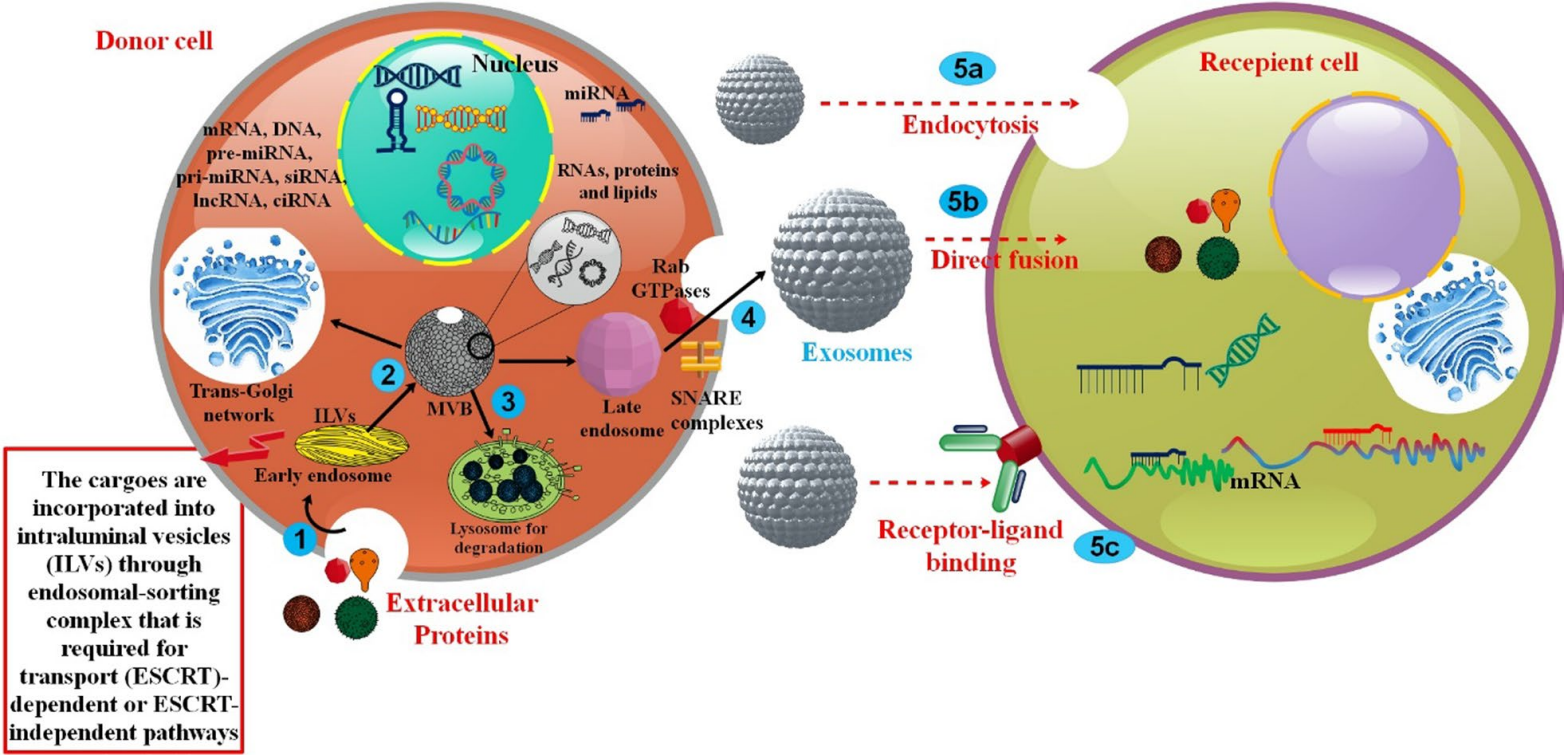
Biogenesis and cargoes of exosomes

The restricted MVB membrane buds inward to create late endosomes, which after that continuously manufacturing exosomes. Large MVBs include intraluminal vesicles (ILVs) formed by invading late endosomal membranes. This involves incorporating proteins into the invaginating membrane and the engulfment and enclosing of cytosolic components inside ILVs. Upon fusing with the plasma membrane, most ILVs become "exosomes" and are discharged into the extracellular space [18, 19]. It has been shown that the ESCRT function is essential for ILV development. Protein ESCRTs 0–III comprise a complex protein machinery that functions as a unit to promote MVB formation, vesicle budding, and the efficient sorting of protein cargo. The ESCRT process begins with ubiquitin-binding subunits of ESCRT-0 recognizing ubiquitinated proteins and sequestering them to distinct regions of the endosomal membrane. ESCRT-III, a protein complex essential for promoting budding processes, is formed when the whole complex interacts with ESCRT-I and -II. After the buds are cleaved to create ILVs, the ESCRT-III complex, powered by the sorting protein Vps4, dissociates from the MVB membrane [20, 21]. The process of a molecular payload being endocytosed by a cell starts the exosome biogenesis pathway. The fundamental function of the early endosome, which is the first vesicle produced by the plasma membrane budding into the cell, is to sort the endocytosed cargo and determine its destination. It is the first stop in the endosomal trafficking route. The cargo from the early endosome may go in three different directions [22]. Although the question of whether ESCRT regulates exosome release remains unresolved, exosomes isolated from several cell types have been shown to contain distinct ESCRT components and ubiquitinated proteins. Furthermore, it has been discovered that the typical exosomal protein Alix, which is linked to many ESCRT proteins (TSG101 and CHMP4), takes involvement in endosomal membrane abscission and budding as well as exosomal cargo selection via its association with syndecan. Based on these findings, a theory was developed linking ESCRT function to exosomal biogenesis [23]. Recycled cargo will relocate to the tubular domains on the periphery of the endosomes, where it will split off and merge with either the recycling endosome's plasma membrane or the Golgi network. The early endosome's core vacuolar areas are where cargo not intended for recycling will gather and commit to the endosomal maturation route, ultimately producing the late endosome. Either fusion with lysosomes and subsequent destruction or fusion with the plasma membrane and exosome release are the two possible outcomes for late endosomes. Changes in the endosomal membrane occur concurrently with the endosomal maturation process, in addition to changes in subcellular location. To facilitate its sorting and downstream movement, the endosome first modifies the composition of its membrane. Ceramides are swapped out for sphingomyelin, and Rab11—involved in late endosome trafficking—is replaced with Rab5, a sign of an early endosome that takes part in delivering vesicles toward the cell center. Second, specific endosomal membrane sections begin to invade and bud out from the cytoplasm into the endosome's intraluminal area when vesicular maturation occurs [22, 24]. Rab31 GTPase activation has been the trigger for membrane budding in these microdomains recently. Because it makes it easier for ceramides to be transferred from the Golgi and endoplasmic reticulum (ER) networks onto early and late endosomes, the ceramide transfer protein (CERT) is essential for ceramide-mediated exosome synthesis and secretion [22, 25, 26] (Fig. 1).

### Composition of exosomes

DNA, mRNA, miRNA, and proteins are only some substances that may be found in exosomes. Studies have attempted to decipher the processes governing RNA loading in exosomes because of their abundant RNA cargo. Exosomes contain several different types of RNA, including miRNA, mRNA, vault RNA, Y-RNA, rRNA, transfer RNA (tRNA), and ribosomal RNA (rRNA) [27].

Usually, there are less than a hundred proteins in a single EV. The number of EVs needed to produce 1 µg of total protein is around ≈109–1010. However, because of their functions in EV biogenesis and protein sorting, some proteins are consistently present in certain EV types, even though protein cargos are varied amongst EVs of the same type. Proteins serve as indicators in clinical diagnostics and EV characterization, allowing for the differentiation of exosomes, microvesicles, and apoptotic bodies. The tetraspanin proteins CD9, CD63, and CD81 are widely distributed on exosome membranes and function in membrane fusion, signal transduction, and protein trafficking; these proteins are hallmarks of exosomes. Exosome biogenesis also involves ALIX, flotillin, and TSG101 [28]. Exosomes also include abundant heat shock proteins (Hsp70 and Hsp90) and cytoskeletal proteins (actin and myosin). These proteins may be utilized to identify and isolate exosomes because they act as markers that set them apart from other extracellular vesicles (EVs) [29].

**Fig. 2:**
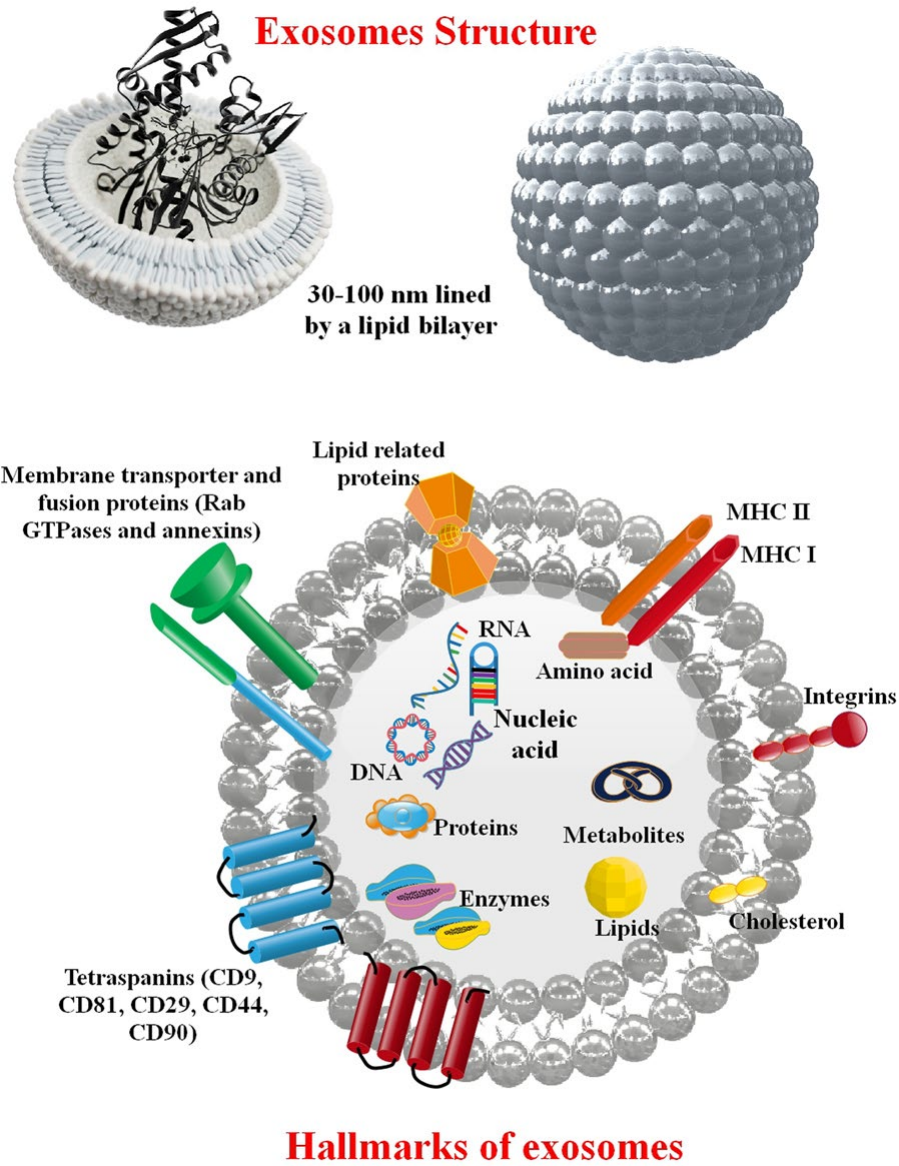
Exosomes, which originate from the plasma membrane, are phospholipid bilayers that include cytoplasm components from the parent cell. The exosomes' makeup is determined by the kind of cell from which they originated, the health status of the parent cell, and the presence or absence of extracellular stimuli. The bulk of exosomes share several proteins, lipids, and miRNAs [33]

Unlike their protein and nucleic acid contents, the lipid composition of EVs is not as well known. Through the use of mass spectrometry, hundreds of lipid species in EV membranes have been characterized. Cholesterol (42.5%), phosphatidylcholine (15.9%), sphingomyelin (12.5%), and their derivatives are the most abundant lipids in platelet-derived exosomes. Exosomes and their parent cells have very different lipid contents, although exosomes from the same cell line have far less variation. The stiffness and resistance to degradation that makes exosomes such efficient transporters of proteins and nucleic acids may be due, in part, to the more significant amount of sphingomyelin, desaturated lipids, and cholesterol in the plasma membrane of exosomes than in their parent cells. Exosome stability may be attributed, in part, to the uneven distribution of lipids between the inner and outer leaflets of the exosomal membrane. In EVs, lipids serve as regulators as well. Another example of lipids' ability to influence inflammation is the anti-inflammatory function of ceramide phosphates in exosome membranes after exposure to secondhand smoking in the bronchoalveolar lavage fluid. Lipids are crucial to the activity of EVs, and a thorough examination of lipids in EVs might reveal new physicochemical features that can be used to distinguish exosomes from other EV subtypes [30–32] (Fig. 2).

## The role of the exosome in viral diseases

**Fig. 3:**
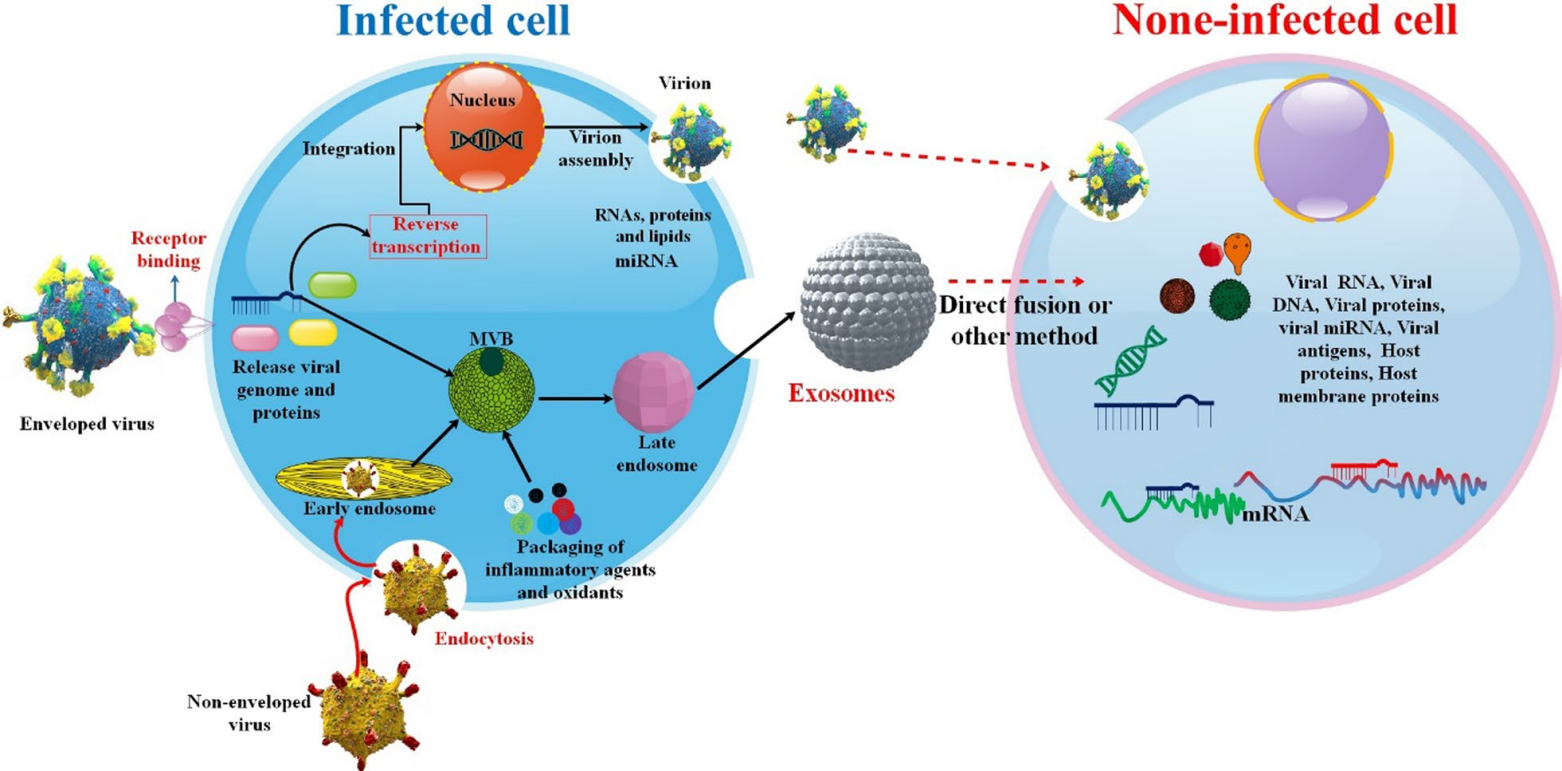
Potential mechanisms of viral transmission by exosomes. EVs play a crucial role in facilitating viral infection through three distinct mechanisms. Firstly, they enable the transfer of cell surface proteins or chemokine co-receptors to null-target cells that do not possess their viral co-receptors. This transfer enhances the susceptibility of these cells to viral infection. Secondly, EVs assist viruses in evading the host immune system, thereby promoting their survival and replication within the host. Lastly, EVs transfer viral components, such as viral proteins and RNAs, to recipient cells. This transfer leads to cytotoxic effects on infected cells and gradually depletes immune cells due to the death of uninfected bystander cells [37]

Exosome release is a fascinating technique that cells use under normal conditions to interact with their environment. In homeostatic settings, exosomes have been shown to transport functional proteins, lipids, and unique sets of RNAs from one cell to another. In theory, this equipment might also aid in disseminating viruses [34]. Furthermore, exosomes have a significant role in endocrinology, inflammation, and cancer and even enable viral infection, making them a promising new clinical diagnostic biomarker. Exosomes have various biological functions and move freely throughout the body, accumulating in various fluids. Therefore, exosomes may soon play a crucial role in clinical diagnosis and therapy as a potential noninvasive liquid biomarker [35]. Exosomes have shown promise as a possible diagnostic and therapeutic tool in clinical settings. Exosomes are an essential element of circulating biomarkers and may be used as a non-invasive diagnostic tool for various disorders [36]. Furthermore, it is crucial to clarify how exosomes exercise their influence on the immune system or increase viral infectivity by making use of their inherent character. Viral replication, infection dissemination, and immune system avoidance begin with the virus hijacking the exosome biogenesis machinery. However, exosomes also play a role in defense by stimulating the innate immune system. In addition, exosomes generated by cells may convey much information about the sick condition, making them a possible diagnostic for identifying viral infections [14] (Fig. 3).

## Exosome isolation methods

Because of the difficulty in detecting exosomes in complicated biological materials, isolation procedures are generally necessary before exosomes can be analyzed in primary or clinical research settings. Exosome production, purity, and integrity might vary depending on the purification process used, and these approaches often need trade-offs between time, money, and effort. The common techniques of differential centrifugation, filtration/size exclusion, and immunoprecipitation have certain limitations. Microvesicles, protein aggregates, lipid droplets, lipoproteins, and other plasma membrane-derived vesicles can contaminate exosome fractions that are isolated using size exclusion techniques (such as volume-excluding polymers precipitation, SEC, and microfluidic sorting) [38]. The current gold standard for isolating exosomes, differential ultracentrifugation, is not only prone to these limitations but also affects the morphological integrity of the isolated exosomes due to the enormous shear pressures it treats them to during the separation process. The isolation of pure exosome samples is further complicated by the discovery of two exosome subpopulations with distinct morphologies and sizes, as well as a new "exomere" category of nonmembranous nanovesicles that can cosegregate. This was discovered in a recent study that used an asymmetric flow field-flow fractionation approach to isolate vesicles by their relative density and hydrodynamic properties. Distinguishing and isolating these exosome subpopulations, however, requires a labor-intensive workflow that necessitates large samples, expensive equipment, and skilled labor, making clinical applications unlikely absent significant methodological improvement [39, 40]. Exosomes may be isolated in sufficient quantities and with adequate purity, thanks to the rapid development of scientific and technological methods. There are a variety of methods for isolating exosomes, and each one makes use of a different property of exosomes, such as their density, shape, size, or surface proteins. The benefits and drawbacks of isolating exosomes might vary greatly depending on the variant within each group [41]. In the following sections follow, we'll go through the many methods that may be used to isolate exosomes.

### Ultracentrifugation-based isolation techniques

Particulate components in a heterogeneous mixture (suspension) will sediment according to their density, size, and shape when the suspension is centrifuged. Denser and/or larger particles tend to fall to the bottom first. The centrifugal force used allows for the progressive separation of particles in a suspension based on the physical parameters of the particles as well as the density and viscosity of the solvent. To generate centrifugal forces as high as 1,000,000 × g, ultracentrifugation is used. Analytical ultracentrifugation and preparative ultracentrifugation are the two main kinds [42, 43].

Preparative ultracentrifugation is a crucial step in isolating exosomes since it is designed to separate minute bioparticles such as viruses, bacteria, subcellular organelles, and EVs. One of the most widely used and documented methods for isolating exosomes, ultracentrifugation is often regarded as the gold standard. An estimated 56% of all exosome isolation methods used in exosome research use ultracentrifugation. Many people find this method appealing since it requires nothing in the way of technical know-how, is cost-effective in the long run (requiring just the purchase of a single ultracentrifuge machine), and may provide satisfactory results in a reasonable amount of time with little to no sample pretreatments. This is why ultracentrifugation-based methods have gained traction in exosome study. Both differential and density gradient ultracentrifugation fall within the category of preparative ultracentrifugation [44, 45]. To separate exosomes from other components in a sample based on their density and size variations, differential ultracentrifugation is often performed using several centrifugation cycles with varying centrifugal pressures and durations. Ultracentrifugation commonly employs centrifugal forces between ~ 100,000 and 120,000 × g. Human plasma or serum is often cleaned to remove big bioparticles before separation begins, and protease inhibitors are added to the sample to prevent the breakdown of exosomal proteins. After each centrifugation run, the supernatant is collected, and the pellet is discarded or resuspended in a suitable medium like phosphate-buffered saline (PBS) for further centrifugation. This process is repeated until the desired number of exosomes has been isolated. After extraction, exosomes are resuspended in a new medium and frozen at − 80°C for further study. Pelleting, or straightforward ultracentrifugation, is a common technique for isolating exosomes [46, 47].

Isopycnic ultracentrifugation and moving-zone ultracentrifugation are two examples of density gradient ultracentrifugation. Isolation of EVs such as exosomes has seen a rise in the utilization of density gradient ultracentrifugation. In density gradient ultracentrifugation, exosomes are separated according to their size, mass, and density in a centrifuge tube filled with a density gradient medium that decreases thickness from bottom to top. A more extended period of ultracentrifugation is performed after a sample is placed in a thin strip on top of a density gradient medium. When a sample is spun, the exosomes and other solutes sink to the bottom of the container at different rates due to the density gradient medium. This results in distinct solute zones. Once the exosomes have been isolated, they may be retrieved with little effort via fraction collection. The separated exosomes are found at the interface of the density gradient layers in a stepped gradient, making their collection easier than in a continuous gradient, which is employed for analytical applications. Density gradient ultracentrifugation has a lower capacity than differential ultracentrifugation because of the restricted load zone [41]. Isopycnic ultracentrifugation involves loading a centrifuge tube with a density gradient medium encompassing the whole range of densities of solutes in a sample. If centrifugation is performed long enough, the density differential between exosomes and all other solutes will cause them to settle into a distinct zone. Exosomes settle after centrifugation in a medium with a density gradient to the isopycnic density level. Once the exosomes have settled into their isopycnic posture, the centrifugal force sharply concentrates them into a zone. It keeps them there, suggesting that isopycnic ultracentrifugation does not involve motion. In the case of self-generating gradient materials like cesium chloride, a sample containing exosomes may instead be equally mixed with a gradient medium. While a density gradient of cesium chloride is created during centrifugation, exosomes settle into an isopycnic posture. The density region of 1.10–1.21 g/ml is where the majority of exosomes are found; hence, this is where they should be extracted. After collecting an aliquot from the desired density range, the sample is ultracentrifuged for a few minutes at ~ 100,000 × g to yield pure exosome pellets, which are then resuspended in PBS for analysis [48].

Exosomes have been linked in many studies to the biogenesis of human immunodeficiency virus type 1 (HIV-1). Exosome fractionation and proteomics have been utilized to effectively identify different exosomal proteins and provide light on the relationships between exosomes and HIV-1 [49]. Due to their shared physicochemical features and distinct interactions, host exosomes and HIV-1 make isolating exosomes from infected cells difficult. HIV-1 virions are 110–128 nm in diameter, whereas exosomes are generally 30 to 100 nm in size. Furthermore, exosomes have a density of 1.13–1.19 g/mL, similar to that of the HIV-1 virus (1.09–1.16 g/mL) [50, 51]. There is a high risk of viral contamination during efforts to purify exosomes utilizing density and size-dependent approaches. Due to their similar densities (1.16 and 1.18 g/ml, respectively), sucrose gradients have also been tried without much success in isolating exosomes from HIV-1 particles. In addition, due to their identical molecular makeup, exosomes and HIV-1 might be difficult to separate biochemically. In addition to using exosomes as a delivery system for viral particles, viruses have been seen hijacking the exosome route [52]. Exosomes have been isolated from in vitro cell cultures and biological fluids by implementing numerous strategies and techniques. A thorough assessment of exosome isolation techniques was conducted using the colorectal cancer cell line LIM1863 as a model organism. These techniques comprised OptiPrep™ density-based separation (DG-Exos), ultracentrifugation (UC-Exos), and immunoaffinity capture employing anti-EpCAM-coated magnetic beads (IAC-Exos). According to electron microscopy, all exosome separation techniques produced 40–150 nm vesicles, and immunoblotting revealed good results for exosome markers (Alix, TSG101, and HSP70). This methodology used label-free spectral counting to assess each method's efficacy in isolating exosomes and a proteomic profiling strategy to determine the protein composition of exosomes. It was shown that the most efficient way to isolate exosomes was to use IAC-Exos, based on the quantity of MS/MS spectra found for exosome markers and proteins related to their biosynthesis, trafficking, and release. When immunoaffinity capture is restricted, however, DG-Exos offers several benefits for exosome isolation (due to antibody availability and appropriateness of exosome markers) [53].

### Size-based techniques

In 1955, Grant H.L. and Colin R.R. developed a method known as SEC to separate solutes with varying molecular weights. This approach involves the passage of an aqueous solution down a column consisting of starch and water. When a liquid sample is passed through a stationary phase composed of porous particles, molecules with varying hydrodynamic radii experience distinct outcomes. Molecules that possess dimensions smaller than the holes of the stationary phase experience a deceleration due to their penetration into the pores. Conversely, molecules of bigger size, unable to access the pores, are compelled to circumvent the porous particles and, exhibit an earlier elution from the column. In the last five decades, significant advancements have been made in enhancing this technique by using a range of refined, permeable substances, including dextran polymer (Sephadex), agarose (Sepharose), and polyacrylamide (Sephacryl or BioGel). Before identifying exosomes, SEC had already seen significant advancements and had been extensively used for the precise separation of bulky molecules or clusters of macromolecules, including proteins, polymers, and diverse liposome particles. The expertise obtained through SEC-based liposome isolation may be simply applied to exosome separation since exosomes exhibit several analogous physical liposome characteristics. For a decade, many commercial SEC kits have been created by firms to isolate exosomes, including qEV (iZON) and PURE-EVs (Hansa Biomed). The preservation of the inherent biological activity of isolated exosomes is a highly desirable characteristic of SEC because of its potential for therapeutic application and functional investigations in exosome-based research. In contrast to ultracentrifugation and filtering techniques, SEC is conducted using passive gravity flow, hence preserving the structural and functional integrity of vesicles [54, 55]. Finally, SEC's contact-free approach (solutes do not interact with the stationary phases) assures zero to low sample loss and a high yield compared to ultrafiltration-based separation. With so many advantages, it's no wonder that SEC-based exosome separation has exploded in popularity in recent years, paving the way for many novel scientific and clinical studies. Not only is this approach appropriate for handling very small volumes of liquid, but it can also be readily scaled up and mechanized for high-throughput synthesis of exosomes. Based on the proven SEC platform and weight-dependent segment and sample collection systems, iZON has developed an automatic exosome isolation system (qEV Automatic Fraction Collector) that enables rapid, accurate, scalable, and automated exosome separation. The SEC approach has several benefits, but it also has certain drawbacks. Recent research found that exosomes generated using the SEC column often showed larger size distribution, notably in the lower size range, indicating the presence of contaminants with sizes comparable to those of exosomes, including protein aggregates and lipoproteins. At the 2013 ISEV conference, Gardiner advocated using a combination of ultrafiltration and SEC to isolate exosomes to remove such pollutants. Ultrafiltration and SEC were later used together by Shu and colleagues to purify cell culture media. They concluded that the combination technique not only resulted in the harvesting of exosomes with much better purity compared to just SEC or ultrafiltration but also retained exosome function. Using ultracentrifugation and SEC, Rood's team created functioning exosomes [56–58].

### Immuno-affinity-based approaches

The discovery that all exosomes share a subset of proteins and receptors opens the door to developing immunoaffinity-based exosome isolation by exploiting the specificity of the binding between these protein markers and their corresponding antibodies (or exosome receptors and their ligands). Exosomes may be captured via immunoaffinity if they contain a target protein or cell membrane component unique to or prominently displayed on the exosome membrane but has no soluble equivalent in the external fluids [59, 60].

Lysosome-associated membrane protein-2B, transmembrane proteins, heat shock proteins, platelet-derived growth factor receptors, fusion proteins (e.g., flotillins, annexins, and GTPases), lipid-related proteins, and phospholipases are just some of the exosome markers that have been documented over the past few decades. Exosome-human CD63 isolation reagent (Thermofisher), Exosome Isolation Kit CD81/CD63 (Miltenyi Biotec), and the Exosome Isolation and Analysis Kit (Abcam) are just a few of the popular exosome isolation products that have been developed using transmembrane proteins like Rab5, CD81, CD63, CD9, CD82, annexin, and Alix for selective exosome isolation [61, 62]. Immunoaffinity capture is a remarkable tool for separating exosomes into distinct populations based on biomarkers. Using an EpCAM (overexpressed on tumor-produced exosomes) antibody-coated magnetic bead technology, researchers were able to successfully isolate tumor-derived exosomes from cell culture media and other clinical samples. Recently, immunoaffinity separation technologies (such as Exosome-Human EpCAM Isolation Reagent, and Thermofisher) have been commercially accessible to isolate specific exosome subpopulations. Naturally, collecting exosomes of a given origin not only makes it easier to investigate their parental cells but also provides vital markers for illness detection (for instance, by identifying EpCAM-positive exosomes to evaluate the presence of EpCAM-related tumors) [58].

### Precipitation methods

The precipitation-based approaches, which include incubating samples with precipitation agents and then low-speed centrifugation or filtration, are quick and efficient ways to purify exosomes. The precipitation agents might be polymers, most frequently polyethylene glycol (PEG), or salt solutions. Initially, the solution seems to be saturated with dissolving polymers, which makes the exosomes less soluble and causes them to precipitate [63]. After showering, the exosomes will be separated by further low-speed centrifugation (1500 × g) or filtering. The generated pellet will then undergo a PBS wash in preparation for the downstream analysis that comes next. Among the many polymers, PEG has drawn the most interest because of its distinct qualities, which include hydrophilicity, cheap cost, and simple precipitation processes. The main disadvantage of the precipitation procedure is the interference of a precipitation agent and membrane fusion of EVs, even if this approach is easy to apply. Another drawback of this procedure is the co-precipitation of non-exosomal particles, such as proteins and polymeric materials, which results in less successful exosome extraction [64]. Weng et al. mentioned previous research and used high-resolution electron microscopy to show the size and form of the retrieved exosome aggregates and to thoroughly describe the PEG-based precipitation strategy’s process [65]. It should be noted that one of the crucial factors that significantly reduces the isolation yield is the pH level of the isolation environment. According to Ban et al., the isolation yield of exosomes was lowest at higher pH values, while it was most significant in acidic environments. Brennan et al. examined many separation methods, such as polymer precipitation, ultracentrifugation, SEC, and density gradient ultracentrifugation (DG UC), to increase the yield of EV isolation. They found that the polymer precipitation method produced the most significant amount of EVs extracted from human blood [64]. An additional precipitation-based method for EV organic solvent precipitation isolation (PROSPR). The primary idea behind this procedure is to precipitate a soluble protein such that the EVs stay in the supernatant using organic solvents, including acetone, chloroform, and trichloroacetic acid (TCA). The supernatant-containing EVs are concentrated using vacuum concentrators or filtering after the proteins have been removed. Another effective isolation technique that is proposed is charge-based precipitation. Since EVs are negatively charged particles, they may separate EVs in plasma, cell culture, and saliva samples by interacting with positively charged particles, primarily protamine. According to Deregibus et al., using PEG and protamine together yields a greater isolation yield than using them individually. As with the other procedures, this method has the advantages of being inexpensive, straightforward, and able to extract intact EVs like precipitation. However, protamine residue may contaminate the final target and necessitate further gel filtering. Furthermore, acetate was used in this study to precipitate EVs by neutralizing negatively charged EVs with acetate by the proposal of charge neutralization, another alternative technique based on precipitation strategy [66].

### Microfluidics-based isolation techniques

Microfluidic-based platforms have been modified recently to indicate uses in the biological applications and diagnosis/treatment fields. Due to its intrinsic qualities, which include laminar flow, minimal sample consumption, simplicity of use, high surface-to-volume ratio, and quick analysis time, microfluidic devices are well suited for separating exosomes with high purity and recovery rates for therapeutic applications. Polydimethylsiloxane (PDMS), polymethyl methacrylate (PMMA), glass, paper, silicon, and metals are among the materials that are used in the fabrication of microfluidic devices; however, PDMS is the material that is most commonly used because of its essential properties, which include transparency, biocompatibility, cost-effectiveness, and flexibility. Microfluidic methods are generally divided into two categories: passive and active. The active approaches need external actuators, such as sound waves, magnetic, and electric fields. Passive techniques, on the other hand, sort particles without the need for external fields. While active separation methods boost the efficiency and throughput of exosome isolation, they also raise the system's complexity and operating costs. Examples of many microfluidic-based isolation techniques are provided, along with information on their advantages and disadvantages [66, 67].

### Filtration

Filtration is a widely used label-free isolation and separation technique, as previously explained. It is also applied to microfluidics, which uses nanoarrays or microchannel-integrated porous membranes to collect exosomes according to size. Membranes are often used in different filtering operations: dead-end and cross-flow (tangential-flow). Researchers used a PDMS microfluidic system to separate exosomes from pure human plasma using tangential and standard flow filtration techniques [68]. To isolate exosomes according to size, a membrane with an 80 nm pore size was used. This study found that using normal flow membrane separation technology caused the membrane to get caked over, which decreased the effectiveness of capturing, raised the pressure drop, and ultimately caused membrane clogging. Conversely, the tangential flow approach demonstrated a high rate of exosome recovery and separation along with a small amount of cake development on the membrane surface. The device was able to function for a more extended amount of time overall because of the usage of an ultrathin silicon nitride membrane, which demonstrated a smaller pressure drop. Additionally, the exosome separation and release efficiency would rise with ultrathin membranes because particles would not be confined inside the membrane’s bulk. A label-free rapid separation method for isolating exosomes from a variety of biofluids, including tears, saliva, plasma, and culture media, was recently published by researchers [69]. Using a different technique, an array of micropillars spaced 900 nm apart could remove bigger particles, including apoptotic bodies and cell debris, in addition to supporting nanowires. Next, exosomes with a size range of 30–200 nm were trapped within the nanowires based on the distance between them. In ten minutes, the isolation experiment was finished without any nanowire deterioration. The porous nanowires were dissolved in the PBS buffer solution for a whole night to retrieve the trapped exosomes. The scientists also said that by using certain antibodies in porous silicon nanowires, it was feasible to improve the device's sensitivity and functioning and investigate the immunoaffinity-based isolation approach. These label-free methods' primary drawback was their lack of specificity, which made it impossible to identify any particles with the same exosome size. Moreover, the membrane and microfluidic device may get clogged with particles once they aggregate on the nanowires or nanoporous membrane [66].

#### Inertial lift force

In microfluidic systems, the inertial lift force is a passive method for isolating the exosomes. Particles are positioned across the microchannels under the inertial force according to the disparity in size and velocity between the fluid and the particles. The scientists sorted the microparticles in the microchannel according to size using a rapid inertial solution exchange (RInSE) approach using an inertial lifting force. Due to their nanoscale size, exosomes were initially cultured with 20-micron polystyrene beads to form a massive complex of exosome beads unaffected by inertial forces. Beads are transferred into the wash buffer solution after being pushed by an inertial lift force toward the middle of the microchannel [70, 71].

#### Viscoelastic flow

Viscous microfluidic devices provide a label-free, size-based method for isolating exosomes. Particles may be sorted by size using the elastic lifting force, which is proportional to the particle volume. One example is the work of Liu et al., who isolated exosomes from cell culture medium and fetal bovine serum (FBS) samples using a high aspect ratio PDMS microfluidic system. The sample and sheath fluids containing a small amount of polyoxyethylene PEO (0.1 wt%PEO) were injected into the microfluidic device through a syringe pump via two inlet holes [72].

#### Deterministic lateral displacement method

One such size-based passive technique is the deterministic lateral displacement (DLD). Here, the microfluidic device is a periodic array of pillar barriers, with the spacing between rows staggered to provide an inclination about the flow direction. There are two modes that the gadget may function in. Larger particles in displacement (bump) mode are moved laterally across an array, whereas smaller particles in zigzag mode follow the streamlines (fluid flow direction). The critical particle diameter is the diameter at which the device switches from operating in zigzag mode to operating in displacement mode, and it may be set by modifying several design factors. Isolation of exosomes from urine and serum samples was accomplished by Smith et al. using a nanoDLD device including 1024 parallel arrays [73]. With a flow rate of 900 μL/hr, the device exhibited excellent throughput and shows promise for use in clinical applications involving exosome isolation. Exosomes ranging in size from 30–200 nm were successfully isolated, and a 50% recovery yield was achieved in both serum and urine samples using this technique. Despite its high-resolution features, this technique may co-isolate other particles, such as viruses and lipoproteins. Since the microscopic particles may become stuck in the tiny spaces in the pillars, clogging is another problem with DLD systems. Furthermore, not only is this approach costly, but the high fluid resistance in DLD-based microfluidic devices limits the separation of large sample volumes. The limits of this approach have been discussed at length in the literature, and there are ways to overcome them [74].

#### Acoustic waves

Using acoustic waves to manipulate cells and bioparticles has improved precision and biocompatibility. The acoustic nano filter is a potential method for isolating exosomes without using any external labels or invasive procedures. The sample is placed in a chamber and subjected to ultrasonic waves; the radiation forces are then given to the particles, which react to the force by their compressibility, size, and density. Because of the acoustic radiation forces, the particles are now moving toward the pressure nodes. The force of Stokes's drag prevents particles from reaching the pressure nodes. Particle volume is directly proportional to the acoustic force, whereas particle diameter directly correlates to the drag force. Due to the increased radiation force big particles encounter and their rapid movement to the pressure nodes, particle separation may occur depending on the particle size distribution. However, the acoustic force and the drag force are almost equal in magnitude in small particles therefore there is slight lateral displacement in them. Adjusting the input power allows for a particle cut-off size proportionate to particle size [75, 76].

#### Non-contact microfluidics

Microfluidic systems based on viscoelastic media flow use the separation concept of non-contact microfluidics, in which the fluid carrying the electric vehicle meets a sheath flow along the microchannel wall. The viscoelasticity of fluids creates an elastic lifting force that pulls exosomes and other extracellular components toward the microchannel's midline in a size-dependent manner. Larger particles eventually reach the centerline, thus achieving the isolation of exosomes. Researchers have also developed a 3D-SiO2 porous chip for quick EV enrichment and detection, which has been used for prostate cancer (PCa) staging in mice and early detection in human PCa patients. According to the findings, non-contact microfluidics may increase the clinical early detection rate. The 3D-SiO2 porous chip may considerably increase biosensing sensitivity by combining nanoscale porosity features with exosome-specific markers [77].

#### Dielectrophoretic and electrophoretic techniques

**Fig. 4:**
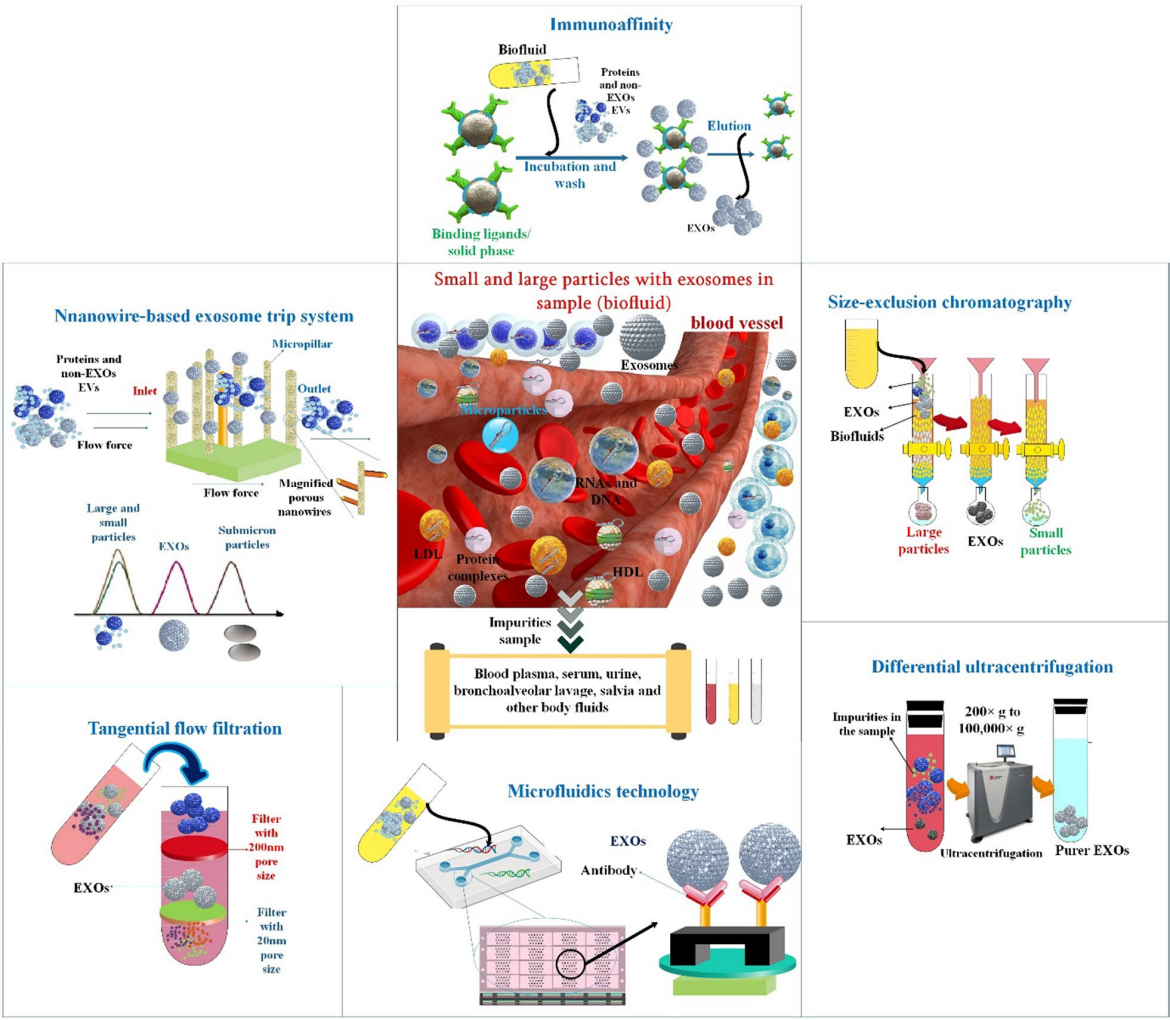
Different strategies for isolating and purifying extracellular material are shown schematically [80]. Both conventional and novel EXO purification techniques. Two of the most used methods for EXO split are size-exclusion chromatography and differential ultracentrifugation. Immunoaffinity absorption uses antibodies that target proteins on the surface of exosomes to separate specific vesicles. The microfluidics (MF) technique uses chips with specialized antibody-interceded connections to capture EXOs effectively. To create a filtrate rich in vesicles with the correct dimensions using ultrafiltration, a filter with a particular pore size is needed [81, 82]

Dielectrophoretic (DEP) describes the polarization and force felt by a particle when it is exposed to a non-uniform electrical field. Particle volume, the absolute permittivity of the particle and solution, and the electrical field intensity gradient value applied via the microchannel all contribute to the DEP force exerted [70]. Negative and positive dielectrophoresis are two of the most common types of DEP separation techniques used today. Particles having a higher degree of polarization relative to the surrounding solution undergo positive dielectrophoresis, in which they migrate to the area of the electrical field with the highest intensity. When the solution is more polarized than the particles, a negative dielectrophoresis occurs, and the particles move toward the weaker electrical field. This means that the size and dielectric characteristics of particles may be used to sort them. Some research has used this technique with success, although it carries the danger of localized heating because of the voltage applied across the microfluidic device. Furthermore, the fluid conductivity requirements of DEP-based approaches are often not compatible with biofluids with physiological osmolarity. Example: Ayala-Mar et al. separated exosomes from a pre-prepared sample using the DEP approach in a PDMS microfluidic system [78]. The plasma sample and exosomes would have to come into touch with the electrodes, which might hurt the bioparticles. A porous hydrogel coating was applied to microelectrodes to overcome this problem. This barrier not only prevented exosomes from damaging themselves on the microelectrode but also reduced the number of bubbles produced by electrolysis. When separating charged particles, the electrophoretic force is superior to the DEP force. Since exosomes are negatively charged, they may be separated using electrophoresis, as was previously explained. Two opposing forces act on charged particles when subjected to an electric field. The particle is sped up by the electric field, but its forward momentum is limited by the drag force. Consequently, over time, the charged particle attains its terminal velocity, at which point the forces of electrical field and drag achieve equilibrium. This equilibrium is contingent upon the particle's charge, size, and the intensity of the electrical field. Notably, Cho et al. have utilized electrophoresis with membrane filtration featuring a pore size of 30 nm to isolate EVs from mouse blood plasma [79] (Fig. 4).

The workings of the exosome trip system based on nanowires. A nanowire-on-micropillar hierarchy structure, similar to SEC-based separation, might be made by imprinting nanowires made of porous silicon onto the walls of the uniformly spaced micropillars. Particles in fluids undergo distinct fates after being introduced to a nanowire-on-micro-pillar tiered structure containing exosomes. The sub-micrometer micropillar array is designed to filter out anything larger than a cell. Submicron-sized particles (such as cell waste) may reach the micropillar interval, but they are blocked from entering the 30–200 nm nanowire interval. Proteins and other small molecules may freely traverse the nanowire gap (Fig. 4). The nanowire forest stops particles between 30 and 200 nm in size (such as exosomes). Size-dependent separation is made possible because particles of different sizes have observably distinct retention times [58].

## Exosomes detection method

**Fig. 5:**
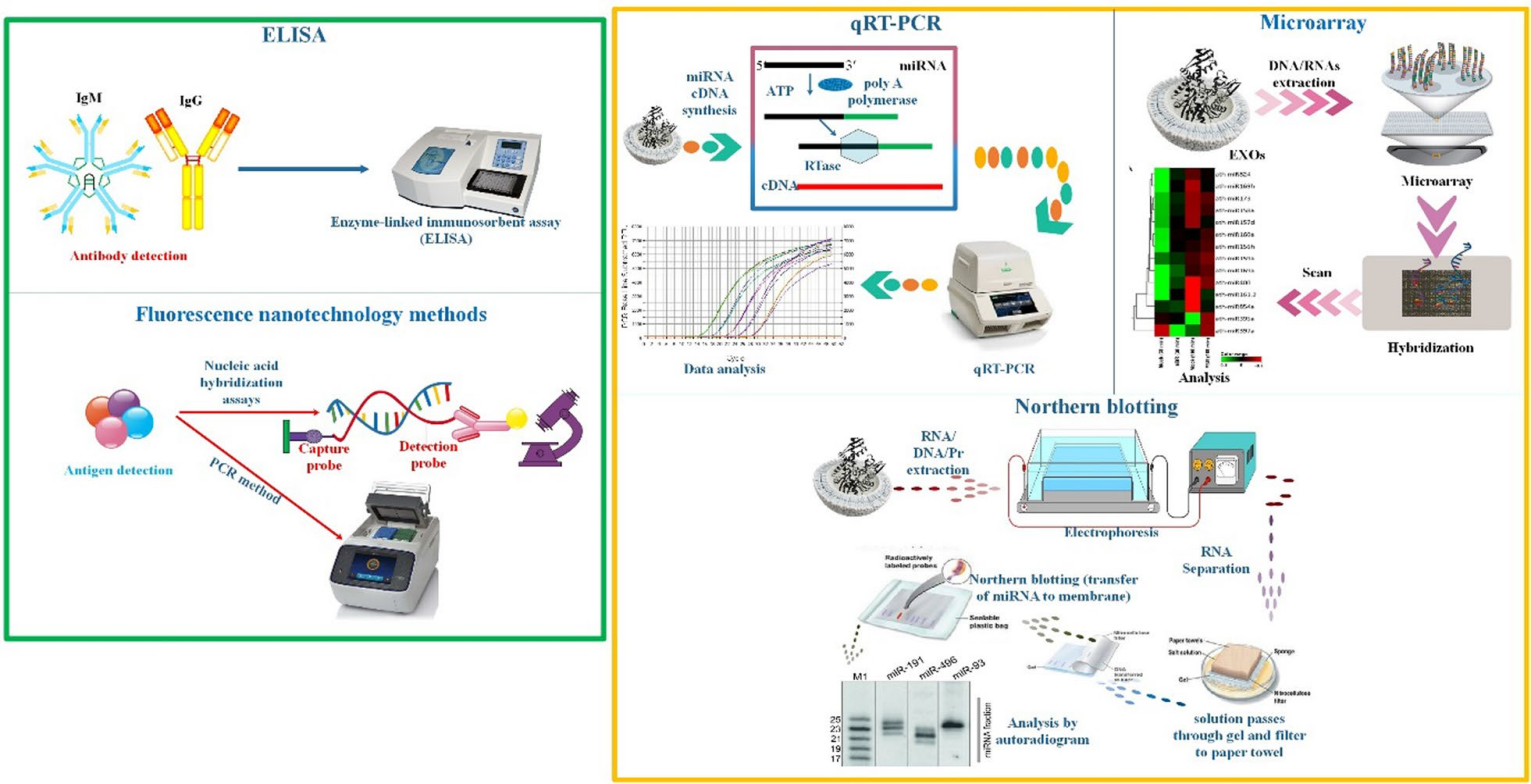
Exosomes detection methods

Finally, mass spectrometry and electron microscopy both indicate the existence of exosome-associated proteins. Due to the minimal manipulation pressures required by sequential filtering, pure and functioning exosomes may be isolated [41]. Enzyme-linked immunosorbent assays (ELISAs), polymerase chain reactions (PCRs), DNA sequencing, and microarray analysis may all identify exosomes that contain protein or nucleic acid biomarkers [83]. Thus, ELISA is a potent instrument since tetraspanins allow for very selective detection and quantification of subsets of EV, such as exosomes. Size and EV content might be estimated with the help of TEM [84]. Remarkably, some proteins are present in exosomes, such as tetraspanins (CD9, CD63, and CD81), heat shock proteins (HSP60, HSP70), and ESCRT-related components (Alix and TSG101). This allows for precise markers to be used in their identification and diagnosis including [85]. Due to its versatility and the fact that it does not need to detect a particular marker, Nanosight NTA has emerged as the gold standard for exosome quantification [86] (Fig. 5).

### Enzyme-linked immunosorbent assay (ELISA)

The ELISA is a standard technique for the detection and measurement of analytes such as proteins, peptides, and antibodies. The Ag-Ab reaction between an antigen and an antibody is the foundation of this method. This chemical process can be reversed, but only if the binding is strong enough to overcome the molecules' natural repulsion from one another [87]. Researchers have been working to develop reliable methodologies for EV quantification in response to rising consumer demand. Thus, ELISA is a potent instrument since tetraspanins allow for very selective detection and quantification of subsets of EV, such as exosomes. TEM may also be used to assess the amount of EVs present and their size. But with the correct reference points, ELISA is the better option. This approach can identify and quantify EV from a particular cellular origin. Still, theoverall concentration of the EV cannot be extrapolated owing to the heterogeneity and subtypes of EV with distinct membrane proteins that may be employed as markers. The expression of CD24 and EpCAM on ovarian cancer cell-derived exosomes demonstrates the potential relevance of EV in cancer diagnosis [88]. Although ELISA kits are already available for detecting and quantifying EVs, they may not always be the best option. Exosome detection and quantification ELISA tests have also been developed in-house. In addition, the ELISA approach has potential use in biomedical research since it may be optimized for the detection of EV obtained from a variety of sources (cell culture and biological fluids such plasma, serum, urine, and cerebrospinal fluid). Potentially valuable for biomedical research, the ELISA approach may be optimized to detect EV in various sample types (cell culture and biological fluids such as plasma, serum, urine, and cerebrospinal fluid) [89].

### Western blotting

Proteins are separated and seen by gel electrophoresis in a process known as western blotting, sometimes called immunoblotting, because of the employment of particular antibodies. Purified exosomes may be identified in EV investigations using this method because of the proteins they express on their outer membrane. Gel electrophoresis is used to classify a protein mixture by molecular weight and species. Each protein then arranges itself into a band upon insertion into a membrane[90].

### Flow cytometry

Quantitative single-cell analysis is possible with flow cytometry. Suspended cells move sequentially, each one surrounded by a thin fluid stream. Cells and fluorescently tagged cellular components are stimulated by a focused laser beam and then generate light. The data is then processed by a computer after being picked up by a series of photodiodes. It is a valuable method for analyzing and characterizing EVs because of its high statistical power and flexibility in analyzing numerous factors. Single vesicles less than 500 nm may not be reliably detected by standard flow cytometers because of their tiny size and low refractive index. This restriction may be circumvented by attaching microbeads to the EV, allowing for semi-quantitative experiments to be conducted. Therefore, standard flow cytometry may investigate EVs to learn more about their biology and potential therapeutic applications. Different EV populations may now be analyzed with greater precision because of the advent of high-resolution flow cytometers. Measurement of EV quantity, size, and phenotyping requires established protocols for both low- and high-resolution flow cytometry, as was the case with AF4 [91, 92].

### Nanoparticle tracking analysis (NTA)

The use of NTA to analyze particle populations and sizes is on the rise. It relies on a charge-coupled device (CCD) camera capturing the scattered light of a laser beam to identify and track individual particles and their Brownian motions. From around 10–20 nm to about 1000–2000 nm, NTA can identify and analyze particles in real-time. The sensitivity of a given piece of equipment is limited mainly by the quantity of light dispersed by the particles and the quality of its optics. NTA works well with samples that are either monodisperse or polydisperse. Therefore, this method may be used to describe various EV subtypes. Additional characteristics, such as zeta potential, the relative intensity of light scattered, or particle concentration, may be measured using NTA [93]. Particle concentrations as low as 106–1010 particles/mL may be studied with this method since each particle is tracked and measured separately. This is helpful since traditional flow cytometry technologies can only identify particles larger than 200–300 nm, meaning that EV cannot be examined directly and must be handled differently before analysis can begin. The size distribution and concentration of EV may be investigated using NTA, making it a popular technique with potential biomedical applications. In pathological states, the size distribution of EV may change. Using the NTA technique, researchers observed that individuals with breast cancer or PCa had significantly greater levels of EV compared to healthy controls [89, 94, 95]. As a result, this method is allowing researchers to learn a lot more about EVs in disease states. This method allows for simultaneous EV measurement and quantification. Several variables may cause NTA to overestimate the average size, and this is especially essential to keep in mind when dealing with EVs. Reasons include a shift in the distribution center to bigger sizes and a decreased sensitivity for smaller sizes (< 50 nm). NTA is the most used technique for characterizing and isolating EVs. It is impossible to phenotype certain forms of EV using standard flow cytometry; however, this is possible using fluorophore-conjugated antibodies. Despite these benefits, existing EV separation approaches cannot sort EVs based on size. Unfortunately, NTA only permits size characterization and not size segregation. As such, asymmetrical-flow field-flow fractionation (AF4) is a method that permits EV size-based fractionation. Although this method is relatively new to the study of EVs, further effort is needed to standardize their analysis so that findings can be reproduced [96, 97].

### Tunable resistive pulse sensing (TRPS)

Like NTA, this technique is a technological tool for characterizing the size and concentration of EV particles. Two fluid cells are separated by a membrane having holes in the nanoscale. When a voltage is given to a nanopore, the passage of particles through the pore disrupts the flow of the current, causing "peaks" or "pulses" to appear in the graph. The intensity of these pulses is related to the particle concentration, and their width is proportional to the particle volume. The size distribution of EVs is very variable, making TRPS characterization difficult. To achieve consistency and improve the parameters while assessing EV size and concentration, Coumans et al. presented a thorough analysis. It has also been claimed that biological fluids may be used directly on this platform, eliminating the requirement for EV isolation in advance [98].

### Lateral flow immunoassay systems

Well-established at the point of care, the lateral-flow immunoassay (LFIA) can increase access to diagnosis, decrease costs, shorten processing times, and improve user-friendliness. To facilitate sample flow, the LFIA's design incorporates several components in a serial, lateral fashion, with a slight overlap connecting each element. These components include the sample pad, conjugate pad, membrane, and absorbent pad. During the procedure, a small amount of sample is placed on the sample pad. A detection reagent (label) conjugated to a target biological component is immobilized on a pad called a "conjugate pad," and the sample makes its way there [99, 100].

In LFIAs, the capture antibody or immobilized antigen is bonded to a membrane (often nitrocellulose) instead of a plastic well (as in ELISAs). Compared to ELISAs, LFIAs are preferable since the whole test may be completed in a single step and a matter of minutes, eliminating the need for lengthy incubation durations and tiresome processes. As with ELISA, the presence of proteins on the EV surface enables their detection by LFIA. Due to their enormous abundance in nearly every cell type, tetraspanins are commonly utilized as exosome biomarkers and are notably abundant in the membrane of exosomes. To detect exosomes from various origins, LFIAs may be created by combining antibodies against these proteins. Due to their diminutive size, exosomes can pass freely through the membrane and into the fluid below, eventually collected by an antibody anchored on a detection line. Additionally, by using various antibodies on various test lines, the LFIA platform may be advanced into a multiple-target assay. This paves the way for identifying EVs with varying surface protein compositions. In ELISA, for instance, a calibration curve determines an EV value. The detection of EVs by LFIA may be significantly affected by variations in protein composition, location, and density of tetraspanins on the exosome surface. Therefore, it is essential to create LFIAs tailored to distinct populations of exosomes [101–103].

### Surface plasmon resonance (SPR)

The phenomenon known as surface plasmon resonance happens when light passes through a glass prism and strikes a metal sheet at the interface of two substances with differing refractive indices. When mass traces, such as proteins or vesicles, are introduced onto the metal film, the refractive index at the metal surface side will alter while it remains unchanged at the prism side. The SPR response may ascertain the analyte's adsorption kinetics once the following reflection is measured. The primary benefit of this method is that it allows the analyte to be measured in real-time. Exosomes are a subset of EVs studied using SPR, which may be used to detect, profile, and measure the particle concentration of these exosomes within 200 nm of the metal film. Additionally, a colorimetric nano-plasmonic assay was created lately to measure the concentration of EV utilizing the SPR phenomenon [89, 104].

### Sensors based on microfluidics and/or magnetic particles

Microfluidics technologies show promise in EV isolation since they allow for the detection and analysis of tiny volume samples. Microfluidics, furthermore, may be integrated with several methods to achieve EV quantification. Magnetic particles, like microfluidics, are developing to not only isolate EVs but also detect and quantify them in mobile systems. Throughout their metabolic cycle, erythrocytes engage in the active elimination of microvesicles (MVs) that are bound to phospholipids. An upward trend in the quantity of these erythrocyte-derived MVs has been documented in preserved blood, indicating their potential utility as a metric for assessing the integrity of blood products. However, the absence of standardized, sensitive MV assays presents a substantial obstacle to integrating MV analyses into clinical environments. Scientists described a novel nanotechnology platform that can detect MV in packed red blood cell (pRBC) units with speed and sensitivity. To enrich MVs directly from pRBC units and designate them with target-specific magnetic nanoparticles, a filter-assisted microfluidic device was devised. The subsequent detection of critical molecular markers (CD44, CD47, CD55) and precise MV quantification were made possible by using a miniaturized nuclear magnetic resonance system. The application of the developed platform enabled longitudinal monitoring of MVs in conserved blood units. The findings of this research demonstrated that MV counts increase with time; therefore, they may function as a reliable indicator of blood aging. Additionally, it was discovered that MVs are capable of producing oxidative stress and consuming nitric oxide. Researchers anticipate that as knowledge of MV biology progresses, the developed platform will contribute to enhanced transfusion safety and blood product quality [105]. To this end, researchers have developed an alternating current electrohydrodynamic method for detecting and quantifying exosomes using a microfluidic device with pairs of electrodes [89, 105, 106]. Circulating exosomes derived from tumors, which are enriched with a collection of tumor antigens, have been identified as a potentially helpful biomarker source for the non-invasive cancer diagnosis. The quantitative identification of exosome tumor markers is both desirable and challenging. Researchers devised a straightforward microfluidic methodology (ExoSearch) that enables the enriched preparation of exosomes from blood plasma to perform in-situ, multiplexed detection utilizing immunomagnetic particles. The continuous-flow design of the ExosSearch device allows the quantitative isolation and release of blood plasma exosomes across a broad volume range of preparation volumes (10 μL to 10 mL). Investigators utilized the ExoSearch chip to perform blood-based ovarian cancer diagnosis by multiplexedly measuring three exosomal tumor markers (CA-125, EpCAM, CD24) in a training set consisting of plasma from ovarian cancer patients. The ExoSearch chip demonstrated comparable diagnostic power to the standard Bradford assay (a.u.c. = 1.0, p = 0.001). Furthermore, the preceding methodology relies on capturing off-chip exosomes with a limited quantity of magnetic beads, which precludes the capability of generating enriched exosomes on a large scale for diverse molecular characterizations after the process. Hence, researchers created the ExoSearch chip, which integrates an in-situ multiplexed exosome immunoassay with on-chip continuous-flow mixing and immunomagnetic separation. In contrast to other microfluidic methods currently in use, the ExoSearch chip has unique characteristics. Firstly, it can process sample volumes ranging from microliters for on-chip analysis to milliliters for variable downstream measurements thanks to its continuous-flow operation. Secondly, it can multiplex the quantification of multiple marker combinations in a single sample at a significantly faster rate (~ 40 min). Lastly, the chip has the potential to become a helpful technology in point-of-care and clinical settings due to its simplicity, cost-effectiveness, and robustness. The ExoSearch chip has made possible a one-step exosome test that may be used to diagnose ovarian cancer. This assay involves measuring a panel of tumor markers in a tiny amount of blood plasma (20 μL). The results demonstrated high diagnostic accuracy and were on par with the traditional Bradford assay [107]. As a result, using microfluidic-based technologies is an exciting approach to EV-related research that may have important therapeutic implications [89, 105, 106].

## The role of exosomes in viral disease diagnosis

In addition, diagnostic and prognostic data may be gleaned from detecting viral RNA, DNA, or virions. Patients' illness states may be evaluated using this data regularly. Developing therapeutic uses for exosomes requires a knowledge of how exosomes boost viral infectivity. Exosomes have been related to several viral illnesses, including acquired immunodeficiency syndrome (AIDS), hepatitis, Ebola, and COVID-19 [14].

Protein and EVs in bodily fluids tend to rise in the presence of certain diseases. This phenomenon has been mined as a noninvasive method for pinpointing the biomarker proteins responsible for a disease's diagnosis. Exosomes' putative biomarker function in illness diagnosis and prognosis has therapeutic implications. Using chemicals found in exosomes as a biomarker in HIV-1 diagnosis or prognosis is a possibility. The HIV-1 patient's urine contained several HIV-1-associated proteins, including Gag, Tat, Vpu, Vpr, and Nef. In addition, neuron-derived exosomes (NDE) may serve as a biomarker for HIV-related cognitive decline. Potential HIV-1 prognostic biomarkers include HMGB1, NF-L, and A proteins [108].

Recuperative plasma exosomes (CPexo) were proposed as a treatment for COVID-19 by Anand et al. Based on the literature demonstrating effective and precise pathogen targeting by exosomes in preclinical research, it was hypothesized that CPexo might provide potential therapeutic effectiveness by functioning as an immunotherapeutic, drug carrier, and diagnostic biomarker [109]. As a result, the protein composition of exosomes is widely known to be altered in illness situations, which may be used as a diagnostic marker of the patient's disease status. Exosomes were studied by Barberies et al. to see whether they may be used as a diagnostic marker for SARS-CoV-2 patients. C-reactive protein (CRP), alpha-1-acid glycoproteins (A1AG1 and A1AG2), and CXCL7 were all found to be considerably higher in critically ill patients than in non-critical COVID-19 patients, whereas CCD34, C4BPA, and GELS were found to be significantly lower. Positive and negative patients might be distinguished by measuring CRP protein levels. Furthermore, it was biased toward treating essential patients differently than routine ones [110]. Similarly, EVs were isolated from people who had a molecular test for SARS-CoV-2, as done by Balbi et al. CD142, a platelet tissue factor (activates the extrinsic route of blood coagulation cascade), was predicted as the most distinct surface protein based on exosome profiling. Patients who tested positive for COVID-19 had elevated CD142 activity, associated with high blood TNF-α levels [111]. Rosell et al. found a similar finding, indicating that the high platelet tissue factor is linked to the morbidity and mortality of COVID-19 [103]. Using CD142 as a prognostic surface antigen may be possible now, thanks to this discovery. TNF, IL-6, and IL-1 were all shown to be present in research conducted by Grifoni et al. Exosomes in the bloodstream are being studied as a possible biomarker for predicting the outcome of a COVID-19 infection [112].

Released exosomes from infected hepatocytes contain diagnostically applicable amounts of viral RNA, core proteins, envelope proteins, and DNA. Researchers have shown that exosomes may store HBV DNA; this means that traditional diagnostic methods may be ineffective when HBV is present. However, the exosome-based diagnostic approach may help predict the likelihood of relapses [113]. Exosomes and miRNA-based approaches have been reported by many research groups to be effective in diagnosing and treating hepatocellular carcinoma (HCC) at an early stage. As an alternative to the insufficient diagnosis and low sensitivity of ultrasound screening and Alpha-fetoprotein (AFP), it was postulated that exosomes combined with miRNA might be used to identify HCC at an early stage. Improved diagnostic sensitivity and efficiency were achieved by combining AFP with miRNA-exosome [114]. To identify HCC recurrence, exosomal miR-718 has been identified as a biomarker in another investigation. Another investigator independently reported a comparable study for HCC diagnosis vs. chronic hepatitis. Patients with chronic hepatitis had elevated levels of miR-18a, miR-221, miR-222, and miR-224 in their serum exosomal microRNAs and serum circulating microRNAs compared to those with chronic hepatitis B (CHB) and liver cirrhosis (LC). Estimating levels of microRNA may help differentiate between HCC, CHB, and LC [14, 115].

### HIV

To better understand how HIV-1 interacts with its host, proteomics has been widely used to identify and quantify proteins in exosomes isolated from HIV-1-infected tissues. Mass spectrometry techniques, with an emphasis on exosome and HIV-1 analysis, will be discussed. When used for diagnosis: Since the standard methods for diagnosing HIV-1 only include looking for antibodies and checking for virion generation, any pathogenic problems that arise during latency may go unnoticed. The development of biomarkers for HIV-1-related issues, such as HIV-1 latency and inflammation, may be facilitated by proteomic profiling of biofluid exosomes from HIV-1-infected individuals. Characterizing the exosomal proteome of biofluids and then analyzing viral protein synthesis is the novel technique proposed here. A vigorous viral infection is often accompanied by exosomes rich in viral proteins. Exosomal proteomics may provide a predictive story of HIV-1/AIDS pathogenesis by linking the physiological function of the biofluid exosomal proteome with pathological consequences. In conclusion, proteomic methods and the development of exosome separation have been critical to understanding the fundamental biology of exosomes, the relationship between exosomes and disease pathophysiology, and the process of creating and evaluating exosome treatments. The breadth of exploratory ventures, however, remains limited; biologically by the limited knowledge of exosome-regulatory mechanisms of packaging and release; and technically by the efficiency of exosome isolation and purification methods. We believe that if these problems could be solved, it would be a huge step forward in our knowledge of the etiology of HIV-1/AIDS and related diseases [49, 116]. HIV-positive patients receiving ART with reduced viral loads and HIV-negative controls had their plasma exosome fractions separated. Electron microscopy, immunoblotting, nanoparticle tracking, and Liquid Chromatography with tandem mass spectrometry (LC–MS-MS) and proteomics were used to characterize exosomes. When compared to controls, HIV-positive individuals had higher levels of plasma exosomes, which were associated with decreases in polyunsaturated fatty acids (PUFA) (Docosahexaenoic acid (DHA), Eicosapentaenoic acid (EPA), and Docosapentaenoic acid (DPA)) and increases in oxidative stress indicators (cystine, oxidized cys-gly). In plasma exosomes, untargeted proteomics identified Notch4, CD9, CD63, CD81, immunological activation (CD14, CRP, HLA-A, HLA-B), oxidative stress (CAT, PRDX1, PRDX2, TXN), and exosome markers (PRDX1, CD63, CD81). HIV-positive individuals had higher levels of exosomal Notch4 than controls, which were associated with indicators of immunological activation. When patient-derived exosomes were added to THP-1 monocytic cells, genes linked to immunological activation and interferon responses were expressed. According to these findings, exosomes from HIV patients receiving ART may include proteins related to oxidative stress and immunological activation, modulate myeloid cell immunity, and have pro-inflammatory and redox effects during pathogenesis [117].

It is noteworthy that the efficacy of sucrose gradients in segregating exosomes from HIV-1 particles is compromised due to their comparable buoyant density and size/diameter. To address this issue, Cantin et al. propose the utilization of iodixanol (OptiPrep™) gradients ranging from 6 to 18% to segregate exosomes from HIV-1 particles and apoptotic vesicles. By employing magnetic particles for immunoisolation, exosomes can be isolated from both culture media and bodily fluids in a quick and uncomplicated manner. An effective isolation technique for exosomes is exosome pull-down based on immunoaffinity; this is possible if a particular exosomal cell surface protein distinguishes the exosome of interest from other membranous particles in the biological matrix [52, 118]. A density gradient from OptiPrep™ was used for exosome purification. In a nutshell, OptiPrep™ (60% w/v) stock solution was diluted with 0.25 M sucrose/10 mM Tris, pH 7.5, to create iodixanol solutions at 40%, 20%, 10%, and 5% w/v, thereby creating a discontinuous iodixanol gradient [119]. Protein identification may also be hampered by interactions between virion components and host proteins in exosomes. Purifying exosomes from HIV-1-infected materials using a velocity gradient based on iodixanol (Optiprep) is one solution to this problem. First, the viruses and exosomes are concentrated and filtered through an ultracentrifuge for 45 min at 100,000 g. The pellet is resuspended in PBS and put on top of an Optiprep density gradient ranging from 6 to 18%. Each layer of the gradient increases in density by 1.2%. After centrifuging the material for 90 min at 250,000 × g, the gradient fractions may be extracted. Exosomes are collected in an 8.4–12% iodixanol gradient, whereas HIV-1 particles are recovered at a 15.6% gradient, indicating a considerable difference in their sedimentation velocities. This technique has been used effectively to isolate exosomes from viral particles. Various techniques are available for isolating exosomes in addition to the ones just discussed. However, in general, researchers need to be able to separate the virions from the exosomes and identify the usual exosomes from other EVs to extract exosomes from HIV-1 infected cells. There is also a need to use techniques that retain the exosomes' chemical and biophysical features with little alteration. The challenge is determining the most appropriate and efficient exosome purification procedure for each research, bearing in mind that it may be necessary to use a combination of techniques [120].

Regarding HIV-1 gene transcription and replication, the Tat is crucial. It has been demonstrated to work as a free protein that leaves cells, and either enters adjacent cells or interacts with the surface receptors of adjacent cells to control gene expression and cellular function. This is the first study to describe the release and absorption of Tat from exosomes. Researchers showed the significant presence of HIV-1 Tat in exosomes derived from Tat-expressing primary astrocytes, Tat-transfected U373.MG and 293 T, and HIV-infected MT4 using an HIV-1 LTR-driven luciferase reporter-based cell assay and Western blotting alone or in combination with an exosome inhibitor, OptiPrep gradient fractionation, and exosome depletion. Further evidence that this novel type of extracellular Tat is physiologically active was provided by the demonstration that Tat linked with exosomes secreted by Tat-expressing astrocytes was able to shorten neurites and kill neurons. Finally, the basic domain's significance in Tat trafficking into exosomes was established by constructing a mutant Tat lacking the basic domain. Despite losing its basic domain, the deletion of Tat's dominant-negative role in Tat-mediated LTR transactivation did not seem to affect Tat trafficking into exosomes. Overall, our findings demonstrate that a sizeable amount of Tat is released and present in exosomes, which may aid in the stability of extracellular Tat and increase the variety of cells it may infect [121].

### SARS-CoV2

SARS-CoV-2, a new coronavirus that emerged at the end of 2019, has been spreading globally and has reached epidemic proportions. Lung tissues are the first to be affected by COVID-19, and these tissues regenerate very slowly. After a lung infection, infected cells undergo massive cytokine activation, making prompt medical attention critical for survival [122]. SARS-CoV-2 diagnostics using exosome-based technologies: a funding opportunity (RFA-OD-20–018), issued in August 2020. The primary objective of this initiative was to investigate the feasibility of employing single EV, exosome, and exosome RNA extraction and analysis to detect the currently circulating SARS-CoV-2 virus and other novel and emerging viruses. The National Institutes of Health (NIH) wanted to make the most of existing investments in exosome separation technology. These cutting-edge methods have already shown their worth in the separation and analysis of exosomes; by applying them to SARS-CoV-2, they may be able to fill in some of the gaps in our present COVID-19 testing and alleviate some of the supply chain problems that plague more conventional methods. Saliva is collected as a biofluid sample in many of the technologies being developed for this initiative, and microfluidics is used to separate the viruses before several other detection methods are used. These technologies may be used for everything from high-complexity laboratory tests to POCs (point-of-care) devices. Saliva as a biofluid for the detection of SARS-CoV-2 emerged early in the pandemic owing to the simplicity of collection and high quantities of virus detected in the oral cavity. However, nasopharyngeal swab (NPS) remains the current gold standard of sample collection for SARS-CoV-2 detection. Saliva has been shown by numerous researchers to be a sensitive technique for detecting SARS-CoV-2, even in asymptomatic and moderate infections [123–126]. For the most part, saliva is used as the biofluid of choice in the technologies being developed for the RADx-Rad Exosome initiative, capitalizing on the higher sensitivity associated with this biofluid and meeting a critical public health requirement for easy and accessible sample collection. One grantee, however, is looking at the feasibility of using biofluids other than saliva to detect SARS-CoV-2. These include feces, plasma, and blood. The microfluidic-based systems being developed for viral separation and isolation are part of the RADx-Rad Exosome initiative. It is the isolation step that sets these technologies apart from current diagnostics, as it allows the isolation of intact viral particles before viral detection, and it was originally developed to isolate and analyze a single EV. The inability of current RNA-based diagnostics (RT-PCR-based assays) to distinguish between infectious virus particles and degraded viral leftovers is a major limitation of these tests. Due to viral shedding, RT-PCR studies show prolonged detection of viral RNA in clinical specimens, but this is not always indicative of the patient's infectious potential. Through an enrichment phase for intact viral particles (and/or exosomes) before viral detection, the technologies developed under the RADx-Rad Exosome initiative directly address this difficulty. Technologies supported by this program provide higher sensitivity and specificity for detecting viruses because of the phase of viral isolation. By isolating the virus first, we can identify SARS-CoV-2 much more quickly than with existing diagnostics and with a far lower detection limit. These methods diverge dramatically from one another and existing diagnostics are in detecting SARS-CoV-2. The RADx-Rad Exosome program funds projects that use a range of non-conventional technologies for SARS-CoV-2 detection, including surface-enhanced Raman spectroscopy (SERS), electrical probe-based detection, loop-mediated isothermal amplification (LAMP), and total internal reflection fluorescence (TIRF) microscopy. Compared to the conventional PCR-based diagnostics for viral detection, they are significantly different. All of the technologies developed under this initiative can identify SARS-CoV-2 and can distinguish between the many SARS-CoV-2 variants because of the detection technologies' specificity. In contrast, the COVID-19 molecular diagnostics currently authorized by the FDA rely mainly on sequence-dependent technology [127–129]. Microfluidics is used in the exosome-based SARS-CoV-2 technologies to achieve separation. Because the chips in microfluidic technologies are disposable, they are suited for POC devices and are often affordable and portable. Large-scale production can be easily accommodated by this technology's format, and recent advancements in design and fabrication have made manufacturing easier. Microfluidics also makes it possible to integrate many features, improving usability and potentially detecting numerous analytes in a single sample [130]. Furthermore, predictive indicators for the severity of COVID-19 illness could be useful for better patient classification and support early clinical choices about COVID-19 treatments. It is reasonable to assume that, in addition to the detection of SARS-CoV-2, technologies developed under this program may also be able to isolate and evaluate exosomes as a source of predictive biomarkers since the original application of the technologies being developed under this RADx-Rad program was to accelerate the development of exRNAs and EVs as potential therapeutics and diagnostics. SARS-CoV-2 virions and EVs may be separated simultaneously thanks to microfluidic systems. Changes in the expression of nucleic acids from viruses and EVs may be evaluated using detection technology. Tissue and organ responses to SARS-CoV-2 infection may result in unique alterations in the molecular signatures and cargo of EVs, which may indicate distinct changes in the course of COVID-19 illness. This is a benefit of these technologies' integrated, multi-parametric design. In addition to detecting SARS-CoV-2, the RADx-Rad exosome-based technologies program would also be able to identify prognostic indicators that may signal a propensity toward developing severe illness or PASC. Collectively, these findings may help shape healthcare policies and provide clues toward a cure for COVID-19. In the end, the SARS-CoV-2 program's efforts to develop multi-parametric and integrated methodologies based on RADx-Rad exosome-based technologies may provide novel methods for detecting the present SARS-CoV-2 virus and other possible future viral pandemics [129, 131].

In a recent study, El-Shennawy et al. found an increase in circulating exosomes (ExoACE2) expressing angiotensin-converting enzyme 2 (ACE2) in the plasma of COVID-19 patients. In this preliminary investigation, we provide a strategy for distinguishing between exosomal populations that include ACE2 and those that do not (non-ExoACE2) based on the microRNA (miRNA) signatures they carry. Six patients had their plasma samples sorted using recombinant biotin-conjugated SARS CoV-2 spike protein with the receptor binding domain (RBD). After isolation, exo-miRNA from both ACE2-positive and ACE2-negative exosome subpopulations were characterized by RT-PCR. Different miRNA expression levels were discovered. Upregulated in ExoACE2 relative to non-ExoACE2 were let-7 g-5p and hsa-miR-4454 + miR-7975, whereas downregulated were hsa-miR-208a-3p and has-miR-323-3p. ExoACE2 exosomes may be isolated using the SARS CoV-2 spike-protein-directed method. Potential biomarkers (such as exo-miRNA) for COVID-19 patients may be more thoroughly characterized if purified [132].

### HPV

This research shows that human papillomavirus (HPV)-associated biomarkers are bundled in salivary exosomes and that isolating and efficiently enriching salivary exosomes might improve diagnostic and prognostic performance for HPV-associated oropharyngeal cancer (HPV-OPC). With an estimated 115,000 new cases each year, OPC is one of the fastest-growing malignancies in Western nations and is mostly attributable to the spread of HPV. Surveillance approaches must be developed to enhance early identification and outcomes due to the rising prevalence of HPV-OPC. Salivary exosomes are a good choice for liquid biopsy in several investigations. Both external and intracellular indicators may identify the type and condition of the source cell since exosomes are produced during the fusing of endosomal membrane compartments and plasma membranes. In recent years, HPV has emerged as the strongest predictor of OPCs, outranking more traditional risk factors. This increase is consistent with a trend toward younger and healthier patients who are more likely to wait until they have obvious regional cervical metastases before seeking medical assistance. Unfortunately, even if people went to the doctor more regularly, clinical examination alone would not be able to detect OPC premalignant lesions in their early stages. That's why it's so important to find ways to diagnose OPC at an early stage [133, 134]. The tumor biomarkers found in saliva may be used for screening for OPCs noninvasively and determining a patient's risk of developing the disease. Droplet digital PCR (ddPCR) analysis of entire saliva from HPV-OPC-positive individuals revealed low levels of pathogenic HPV16 DNA, as reported by Wang et al. Only 40% of the samples tested positive for HPV16 DNA. Regarding molecular targets in tumors, exosomes offer a rich vesicular supply. To diagnose HPV-OPC early, it may be possible to isolate tumor-specific exosomes from saliva and determine their molecular markers. It was expected that HPV16 DNA sequences in HPV-OPC patients would be packed in salivary exosomes and that exosome isolation would improve the detection of HPV16 DNA, however, no evidence of HPV DNA in exosomes has yet been observed [135]. Differential centrifugation and immunological capture, two common isolation methods, demand a lot of sample volume, take a long time, and provide impure and low-yield results. However, typical procedures may potentially jeopardize the purity of isolated exosomes due to the high centrifugal forces and/or several washing stages that are usually necessary. Exosome separation techniques are made more difficult by variations in saliva's physical characteristics owing to variables including health and stimulation during collection. Saliva, for instance, has a viscosity between 1.10 and 2.30 mPa · s, whereas plasma has a much more consistent 1.10 and 1.30 mPa · s. The isolation technique for salivary exosomes for liquid biopsies has to achieve steady performance on samples with drastically varied physical characteristics, in addition to having high yield, high purity, and high biocompatibility [136]. To separate exosomes from raw blood samples, researchers have developed an acoustofluidic platform (a combination of acoustics and microfluidics) that employs standing surface acoustic waves (SAW). Exosome separation with excellent purity and yield has been achieved using an acoustofluidic platform. Isolated saliva samples include high-purity exosomes since the particles contain known exosomal protein biomarkers and have the anticipated exosomal size and form. TEM scans indicated that isolated particles did not clump together or burst, suggesting that the exosomes maintained their structural integrity. Exosomal miRNA was quantified using ddPCR for miRNAs, and the results showed that the quantity of miRNA in acoustofluidic-separated samples was more than in the sample isolated by differential centrifugation. Researchers believe that the increased yield results from the consistent and excellent quality of acoustofluidic exosome separation. Differential centrifugation, on the other hand, necessitates the removal of supernatant at many stages, which might result in the accidental loss of exosomes. Furthermore, pellets containing exosomes are hard to see and manually retrieve when processing small-volume samples by differential centrifugation. This acoustofluidic technique would greatly improve the development of a salivary exosome-based liquid biopsy due to the various sources of exosomes in OPC patients' saliva and the often modest sample volume [134]. Due to the importance of viscosity-induced drag force during size-based separation of exosomes, the outcomes of isolating exosomes from different saliva samples might be very variable. Exosome separation from saliva samples of varying physical properties, such as viscosity, was studied and refined using an acoustofluidic platform. It was also shown that the detection rate of HPV16 DNA in patients with HPV-OPC may be increased by the separation of exosomes. Exosomes recovered from the saliva of patients with HPV-associated oral squamous cell carcinoma showed 80% concordance with HPV16-positive tissues/biopsies, demonstrating the efficacy of our high-yield exosome separation technology. Overall, the results showed that high-purity and high-yield salivary exosome separation is possible using the acoustofluidic platform in salivary exosome-based liquid biopsy applications. Salivary exosome separation may improve HPV16 DNA detection since these exosomes include HPV16 DNA sequences from HPV-OPC patients [76, 137].

The interactions between the host immune system and HPV( +) and HPV( −) head and neck cancer (HNC) cells are poorly known. We have discovered molecular and functional distinctions between exosomes generated by HPV( +) and HPV( −) cells, indicating that the genetic contents of exosomes may provide new biomarkers in HPV-associated HNCs. Three HPV( +) and two HPV(-) HNC cell line supernatants were used to extract exosomes using SEC. The mRNA and miRNA contents of paired cell lysates and exosomes were measured by qRT-PCR and nanostring analysis, respectively. HPV( +) and HPV( −) cells had different mRNA profiles, with EGFR, TP53, and HSPA1A/B overexpressed in the former and IL6, FAS, and DPP4 in the latter. The mRNA profiles of exosomes carrying HPV( +) or HPV( −) were similar to those of the parent cells. Eight miRs were expressed in HPV( −) cells compared to 14 miRs in HPV( +) cells, according to miR expression patterns in cell lysates. Only HPV( +) exosomes expressed miR-205-5p, whereas only HPV( −) exosomes showed evidence of miR-1972. It was shown by researchers that the mRNA expression patterns of the parent cells were replicated in HPV( +) and HPV( −) exosomes. miR-205-5p in HPV( +) and miR-1972 in HPV( −) exosomes emerge as possible distinguishing HPV-associated biomarkers. The expression of miRs was depending on the HPV status [138].

### HBV

The unrelated hepatitis viruses A, B, C, and E are the source of liver-related illnesses such as LC and HCC, as well as acute and chronic hepatitis. RNA viruses belonging to the Flaviviridae and Picornaviridae families are hepatitis A viruses (HAV) and HCV, respectively. Hepadnaviridae is a family of viruses that includes HBV. According to Shearer et al. (2018), 3.9% of people worldwide have HBV. 399,000 individuals worldwide pass away from cirrhosis and HCC, while 142 million people worldwide have a chronic hepatitis C infection. Exosomes change in composition due to pathophysiological difficulties, and this may be used to learn more about the condition. Viral DNA, envelope protein, core proteins, and RNA are expressed in the exosomes produced from hepatocytes infected with the virus. These components may be used in diagnostic procedures. There have been studies that point to the exosomes as a possible source of HBV DNA; in these situations, traditional diagnostic techniques may not be effective. Nevertheless, the exosome-based diagnostic method may help identify recurrence risks [113].

The purpose of this research was to investigate the relationship between serum biochemical markers of HBV infection and the sensitivity of exosomal HBV-DNA levels. Globally, there are around 292 million HBV-positive individuals; the largest infection rates are seen in Africa and the Western Pacific area, where > 6% of persons are affected. About 6% of Chinese people are HBV carriers, making China a particularly vulnerable nation. Currently, the HBV-DNA nucleic acid test, HBV serum marker test, liver function test, and other auxiliary tests are the primary methods used to diagnose hepatitis B. According to the most recent edition of the European HBV treatment recommendations, liver function markers such as alanine aminotransferase (ALT) and HBV-DNA must be increased for the treatment requirements to be satisfied. A liver biopsy is required to identify low levels of HBV-DNA replication; however, this procedure is invasive, difficult for patients to accept, and cannot be performed often to track the effectiveness of therapy [139, 140]. Exosomes maintain their stability in bodily fluids and are not readily diluted by blood. Thus, keeping an eye on the amount of HBV-DNA in exosomes may provide a reliable and up-to-date picture of the disease's onset and progression, allowing for the determination of therapy to avert liver cirrhosis and cancer. In a study, researchers suggest that when a patient’s serum HBV-DNA is negative, exosomal HBV-DNA can accurately reflect the level of HBV replication in vivo. This investigation researched exosomal HBV-DNA levels in chronic HBV infection (CHB). Exosomal HBV-DNA was detected in individuals with CHB who tested negative for serum HBV-DNA; this allowed for the tracking of therapy effects. Patients who test negative for serum HBV-DNA but have a strong suspicion of HBV infection may benefit from using exosomal HBV-DNA. Not Taking The size and quantity of serum exosomes were measured by NTA using a particle size analyzer (Particle Metrix GmbH, Ammersee, Germany) [141]. As important clinical markers, serum HBV-DNA and ALT are often utilized to identify and assess CHB patients. When ALT elevation and serum HBV-DNA are present, antiviral treatment has the highest chance of working. However, some individuals have normal ALT and low-elevated HBV-DNA, making it challenging to schedule therapy for them. The degree of the liver lesions may not be fully indicated by these signs, in which case a liver biopsy is necessary to determine the best course of therapy. Exosomes are essential for intercellular communication and may be used as prognostic and diagnostic markers for several illnesses. It should be mentioned that no relationship was found between the amounts of HBV-DNA in serum and exosomes and ALT, a crucial biochemical marker of liver impairment. Thus, tracking the amount of HBV-DNA in exosomes may help select therapy to stop the illness from developing into LC and cancer and immediately and adequately represent the onset and progression of the disease [141–144].

Exosomes and early-stage miRNA-based methods have been reported by several different groups in HCC. To address the insufficient diagnosis and low sensitivity of AFP and ultrasound screening, combining exosomes with miRNA was suggested as an early-stage detection strategy for HCC. The sensitivity and effectiveness of the diagnostic were increased when AFP and miRNA-exosome were combined. Exosomal miR-718 was identified in a different investigation as a biomarker for HCC recurrence diagnosis. An analogous study about the identification of HCC in contrast to chronic hepatitis was reported by another lone researcher. When compared to CHB and LC patients, the samples taken from the chronic hepatitis patients and tested for serum exosomal microRNAs and serum circulating microRNAs indicated substantially more significant levels of miR-18a, miR-221, miR-222, and miR-224. The estimate of microRNA may help differentiate HCC from CHB and LC [14].

### HCV

HCV is spread directly from cell to cell and by cell-free viruses. An additional mechanism to take into account is indirect cell-to-cell communication. In the past, exosome-like structures have been found in the plasma of HCV-infected individuals and the supernatant of HCV-SGR cells. These findings include the identification of HCV viral RNA. Exosomes from HCV-infected Huh7.5 cells and HCV-infected individuals were shown to contain HCV RNA, which caused primary human hepatocytes to become actively infected (PHH) [145, 146]. To study the ability of exosomes isolated from Huh7.5 cells infected with HCV J6/JFH-1 and blood from patients with HCV infection to mediate active transmission, researchers first required to isolate exosomes from free HCV effectively. Exosomes were originally isolated using Exoquick after successive filtering (1 µm, 0.44 µm, and 0.22 µm) of supernatants. Immuno-magnetic selection using CD63, a selection marker of exosomes, was used to purify Exoquick-isolated exosomes further and remove other microparticles or free HCV contamination. Since more exosomes were collected with the Exoquick-CD63 immuno-magnetic selection approach than with ultracentrifugation-CD63 immuno-selection, researchers continued our investigations using this protocol. Researchers found that in HCV J6/JFH-1 infected Huh7.5 cell supernatants, there were almost seven times as many free HCV viral particles as there were exosome particles in the same volume, and in HCV infected patient serum samples, the ratio was about four times as high. J6/JFH-1 infected Huh7.5 cell supernatants, and HCV patient serum had larger quantities of HCV viral copies in the free virus fraction than in exosomes. The TEM analysis of purified exosomes revealed that they were vesicular in form and ranged in size from 50 to 100 nm. HCV exosomes from Huh7.5 cell culture supernatants and HCV exosomes from patient serum were found to have similar histogram size plots after further examination using NanoSight [147].

Seven exosomal miRNAs, including 10b-5p, 221-3p, 223-3p, 10b-5p, 221-3p, 223-3p, and 21–5, were identified by Ghosh and colleagues in 2020 as sensitivity indicators for the early identification of HCC independent of HCV origin, particularly HCCs linked with low AFP. According to research, exosomes taken from the sera of patients with HCV-related HCC had the greatest expression of lncRNA-HEIH, followed by exosomes produced from HCV-induced cirrhosis, and finally, exosomes derived from persons with chronic HCV infection, which had the lowest expression. Potential HCV-related HCC may be evaluated using LncRNA-HEIH levels in the serum and exosomes. Matboli et al. found that exosomes isolated from HCC patients' sera express more lncRNA-RP11-583F2.2 than exosomes from HCV patients or healthy controls. This research shows that exosomal lncRNA-RP11-583F2.2 may be used as a biomarker for the diagnosis and prognosis of HCC. Diseases associated with HCV may be diagnosed with the use of exosomes [148].

### Herpes virus

Herpes simplex keratitis (HSK) is the most prevalent form of infectious keratitis despite being a dangerous condition with a high recurrence rate. Herpes simplex virus type 1 (HSV-1) is the most common culprit. It is unclear how HSV-1 is transmitted in HSK. Exosomes have been shown in many studies to have a role in the intercellular communication process between infected cells. However, the exosomal route is seldom cited for HSV-1 transmission in HSK. This study aims to investigate the relationship between the spread of HSV-1 and tear exosomes in recurrent HSK. Fifty-nine people's tears were analyzed in this research. By ultracentrifugation, researchers could isolate tear exosomes, which were subsequently identified by silver staining and western blotting. The tear fluids did have a higher concentration of exosomes. The sizes of the collected exosomes are typical, which is in line with previous studies. Exosome biomarkers might be found in tears. Human corneal epithelial cells (HCEC) could rapidly and efficiently uptake labeled exosomes. HSK indicators were identified in infected cells by western blotting after cellular absorption. Recurrent HSK suggests that HSV-1 is latent in tear exosomes and that they may play a role in HSV-1 transmission. This research also confirms the exosomal route as a viable HSV-1 gene transfer, opening up new avenues for therapeutic intervention and therapy of recurrent HSK [149].

The varicella-zoster virus (VZV) is one of eight herpes viruses that may infect humans. An infection with the VZV may produce either varicella ('chickenpox') or herpes zoster ('shingles'). The rash associated with VZV infection results from a systemic inflammatory response. A zoster virus infection, or herpes zoster, is a skin sickness brought on by the spontaneous activation of dormant VZV. Because exosomes are created by the body and include proteins involved in blood clotting, having shingles increases your risk of having a stroke. Researchers also found that the exosomes of shingles patients continued to show larger levels of these proteins even 3 months after the first rash. Researchers determined that the contents of these exosomes may induce clotting by exposing healthy platelets to exosomes from shingles patients or healthy persons. A clotting-like reaction occurred when platelets were exposed to shingles exosomes, with the platelets fusing with other blood cells. These long-term studies will also examine exosomes for their potential as a biomarker for monitoring stroke risk after shingles [150].

### Other viruses

Microenvironmental changes are induced in response to exosomes released by Epstein–Barr virus (EBV)-infected cells, which include viral miRNAs and proteins that stimulate target cell production of cytokines and chemokines. Additionally, the equilibrium between the lytic and latent cycles is thought to have exosomes, which are crucial for viral persistence and the pathogenesis of EBV-related malignancies. However, cell-free EBV DNA demonstrates a high sensitivity and specificity in patients with EBV-associated gastric cancer (EBVaGC), and there has been no article so far proving the diagnostic use of exosomes in EBVaGC. Clinical use is anticipated due to the correlation between changes in plasma EBV DNA and the clinical course. Several studies have linked oncoproteins and miRNAs related to EBV with EBVaGC prognosis. Even inside exosomes, LMP2A and EBNA1 are believed to be related to chemotherapy response and potential biomarkers for the diagnosis and prognosis of EBVaGC [151]. Overexpression of PD-L1/PD-L2 is one of the molecular markers of EBVaGC that has been linked to a positive response to immunotherapy. The specific mechanism of PD-L1 overexpression in EBVaGC remains unknown, however, it is assumed to include the JAK2/STAT1/IRF-1 pathway, the PIAS3/pSTAT3 pathway caused by miR-BART5-5p, and EBV miR-BART11 and EBV-BART17-3p. The involvement of exosomal PD-L1 in EBVaGC should get the same level of attention it has received as a prospective diagnostic and therapeutic target in other malignancies [151–154].

The Ebola virus (EBOV) is the most virulent known disease, with a high clinical fatality of 25–90% and an average Case Fatality Rate (CFR) of 50%. It is an enveloped RNA virus from the family Filoviridae. Ebola virus disease, often known as Ebola hemorrhagic fever, is a deadly illness caused by EBOV. There were 11,323 fatalities and 28,646 new infections during the last Ebola epidemic in West Africa (2014–2016), with a CFR of 46.0% [155]. Virus-specific nuclear proteins, such as LMP1 and EBNA2, and cell membrane-specific proteins are expressed in exosomes released from EBV-infected cells. Given the importance of exosomes in viral persistence and host propagation, they must be considered when designing diagnostic or prognostic biomarkers and therapeutic approaches to cancer. Serum and saliva samples from individuals with nasopharyngeal carcinoma (NPC) were analyzed by Houali et al., who identified the LMP1 and BARF1 oncoproteins as possible diagnostic markers. Exosomes are present in the segregated and secreted bodily fluids, saliva, and serum. NPCs were shown to have exosome-like vesicles with viral oncoproteins. Extranodal NK/T-cell lymphoma, nasal type, is unique from other forms of lymphoma, and the LMP1-LMP2a mRNA was shown to be a possible predictive biomarker. Proteins including LMP1, LMP2a, BARF1, HIF1, EBV DNA, EBV miRNA, EBV mRNA, and growth factors have all been extracted from EBV-derived exosomes and have been studied for their potential roles in prognosis and diagnosis [14, 156, 157].

Chickens, geese, and ducks are the primary targets of the Newcastle disease virus (NDV). The severe pathology and high mortality seen in susceptible hosts after infection with the virulent NDV strain is a major setback for the chicken industry. Scientists in this work used a newly discovered protein A/G-correlated technique to isolate the exosome (NDV Ex) associated with the Newcastle disease virus. Both GW4869-mediated deprivation and exosomal replenishment demonstrated that these exosomes increased NDV proliferation. More work revealed that the NP protein may be transmitted to DF-1 cells through exosomes, and the detection of viral structural proteins NP and F in the NDV Ex provided more support for this notion. The NP protein found within cells has properties that encourage viral propagation and inhibit cytokine production. Thus, exosomes are produced during an NDV infection and are crucial to the progression of the infection due to their ability to transport viral NP protein [158] (Table 1).

**Table 1:** Use of exosomes in the detection of the viral infection

Viral infection	Exosome isolation method	Exosomes detection method	Explain	References
HIV-1	Ultracentrifugation	Western blotting	Regarding HIV-1 gene transcription and replication, the Tat is crucial. Overall, our findings demonstrate that a sizeable amount of Tat is released and present in exosomes, which may aid in the stability of extracellular Tat and increase the variety of cells it may infect	[121]
SARS-CoV-2	Microfluidics	RADx-Rad exosome-based technologies	The RADx-Rad program was to accelerate the development of exRNAs and EVs as potential therapeutics and diagnostics. In addition to detecting SARS-CoV-2, the RADx-Rad exosome-based technologies program would also be able to identify prognostic indicators that may signal a propensity toward developing severe illness or PASC	[129, 131]
HPV-OPC	Acoustofluidic platform	Droplet digital PCR (ddPCR)	Isolated saliva samples include high-purity exosomes since the particles contain known exosomal protein biomarkers and have the anticipated exosomal size and form. Exosomal miRNA was quantified using ddPCR for miRNAs, and the results showed that the quantity of miRNA in acoustofluidic-separated samples was more than in the sample isolated by differential centrifugation	[134]
HBV	ExoQuick-TC exosome extraction kit	Nanoparticle tracking analysis (NTA)	Patients who test negative for serum HBV-DNA but have a strong suspicion of HBV infection may benefit from using exosomal HBV-DNA. Thus, tracking the amount of HBV-DNA in exosomes may help select therapy to stop the illness from developing into LC and cancer and immediately and properly represent the onset and progression of the disease	[121]
HCV	D63 immuno-magnetic separation	Western blotting (for CD9 and CD81), electron microscopy, and NTA	Researchers found that in HCV J6/JFH-1 infected Huh7.5 cell supernatants, there were almost seven times as many free HCV viral particles as there were exosome particles in the same volume, and in HCV infected patient serum samples, the ratio was about four times as high	[147]
HSV-1	Ultracentrifugation	Western blotting	Recurrent HSK suggests that HSV-1 is latent in tear exosomes and that they may play a role in HSV-1 transmission. This research also confirms the exosomal route as a viable HSV-1 gene transfer, opening up new avenues for therapeutic intervention and therapy of recurrent HSK	[149]

## Future perspectives

Exosomes have been shown to have a variety of biological uses, but one of the most promising is as biomarkers in clinical diagnosis. Due to their exceptional stability, exosomal biomarkers provide the same or even greater specificity and sensitivity than biomarkers discovered in traditional collections like serum or urine. Clinical applications would benefit greatly from using exosomal biomarkers extracted from readily available biofluids like saliva. The recent improvements in exosome separation technology have not only made exosome research easier but also reduced the cost of exosomal diagnostics. Exosomal lipids have been found to have diagnostic potential in addition to exosomal proteins and RNAs [159]. Multiple exosomal miRNAs and proteins have been found with the potential to serve as biomarkers for the diagnosis, prognosis, and therapeutic response prediction of cancer. However, evaluating exosomes' clinical utility is complicated because various studies' data cannot be compared since they were collected using different methodologies [1, 160]. There is an excellent potential for exosomes to serve as biomarkers for early diagnosis, illness staging, and therapy monitoring since they are released abundantly by most cells and contain membrane and cytosolic components that might reflect the physiologic condition of their parent cells. Although exosomes may be helpful in conventional diagnostic or prognostic applications, the absence of quick and sensitive tests that may harness their biological information has limited their use [40]. Exosome research is exploding as a promising biomarker for disease diagnosis, prognosis, and treatment monitoring (in conditions like cancer, infectious diseases, and neurodegenerative diseases). This highlights the need for susceptible, repeatable, and scalable techniques to be developed to analyze and apply exosomes in clinically relevant applications [40]. Additionally, the presence of viral RNA, DNA, or virions is found, which offers diagnostic or prognostic information. To evaluate the patients' illness states, the data may be routinely reviewed. Understanding how exosomes increase viral infectivity will help create exosomes for use in therapeutic settings. Exosomes have been connected to several viral illnesses, including AIDS, hepatitis, Ebola, COVID-19, and EBV [14]. Because of their cell-specific origin, simple accessibility, and non-invasive nature, exosomes are a promising candidate for use as diagnostic biomarkers. The exosomes may enhance the sensitivity and accuracy of diagnostic markers when used in conjunction with currently available diagnostic techniques. The whole genomic DNA and mutations found in the original cells are represented by the exosome DNA, which may also be used to forecast illnesses brought on by mutations [161].

In exosome-based applications, methods for isolating and purifying exosomes provide the greatest hurdle. The recovery and specificity of each approach differ, but their use depends upon the application area. Standardized methods are required for the separation and refinement of exosomes. The methods would be highly recoverable, high throughput, standardized, feasible, and easily replicable [162, 163].

## Conclusion

Exosomes are useful diagnostic and prognostic indicators since their contents are derived from the cells that produced them. Recent research has shown that exosomes play a significant role in the progression of viral infection. Exosomes promote viral infection and antiviral immunity and are critical viral transmission vehicles. The exosomes' heterogeneity results from the wide range of cells and products from which they are derived; this in turn, allows for a wide range of biological roles and enough room for artificial modification. Researchers' attention has switched from fundamental research to clinical application, particularly the development of viral infection detection techniques, due to advances in isolation and purification technology and detection methods. When it comes to detecting exosomes, electrochemical nanosensors have shown to be quite useful due to their low maintenance requirements, excellent precision, and consistent reproducibility. Developing therapeutic uses for exosomes requires a deeper understanding of exosome biology. To further investigate whether exosomes promote viral propagation or induce immune protection, it may be useful to track exosomal composition throughout infection.

## Data Availability

Not applicable.
